# Exploring the Interactions between Plant Proanthocyanidins and Thiabendazole: Insights from Isothermal Titration Calorimetry

**DOI:** 10.3390/molecules29153492

**Published:** 2024-07-25

**Authors:** Mimosa Sillanpää, Marica T. Engström, Petri Tähtinen, Rebecca J. Green, Jarmo Käpylä, Anu Näreaho, Maarit Karonen

**Affiliations:** 1 Department of Chemistry, University of Turku, FI-20014 Turku, Finland; peppe@utu.fi (P.T.); maarit.karonen@utu.fi (M.K.); 2 Institute of Biomedicine, University of Turku, FI-20014 Turku, Finland; mtengs@utu.fi; 3 School of Chemistry, Food and Pharmacy, University of Reading, Whiteknights, P.O. Box 224, Reading RG6 6AP, UK; rebecca.green@reading.ac.uk; 4 Department of Life Technologies, University of Turku, FI-20014 Turku, Finland; jakapy@utu.fi; 5 Department of Veterinary Biosciences, University of Helsinki, FI-00014 Helsinki, Finland; anu.nareaho@helsinki.fi

**Keywords:** condensed tannin, interactions, isothermal titration calorimetry, mean degree of polymerization, proanthocyanidin, procyanidin, prodelphinidin, tannin–anthelmintic interactions, tannin, thiabendazole

## Abstract

Anthelmintic resistance in gastrointestinal nematodes produces substantial challenges to agriculture, and new strategies for nematode control in livestock animals are called for. Natural compounds, including tannins, with proven anthelmintic activity could be a functional option as structurally diverse complementary compounds to be used alongside commercial anthelmintics. However, the dual use of two anthelmintic components requires an understanding of the pharmacological effects of the combination, while information concerning the interactions between plant-based polyphenols and commercial anthelmintics is scarce. We studied the direct interactions of proanthocyanidins (PAs, syn. condensed tannins) and a commercial anthelmintic thiabendazole, as a model substance of benzimidazoles, by isothermal titration calorimetry (ITC). Our results show evidence of a direct interaction of an exothermic nature with observed enthalpy changes ranging from 0 to −30 kJ/mol. The strength of the interaction between PAs and thiabendazole is mediated by structural characteristics of the PAs with the strongest positive correlation originating from the presence of galloyl groups and the increased degree of polymerization.

## 1. Introduction

**Figure 1:**
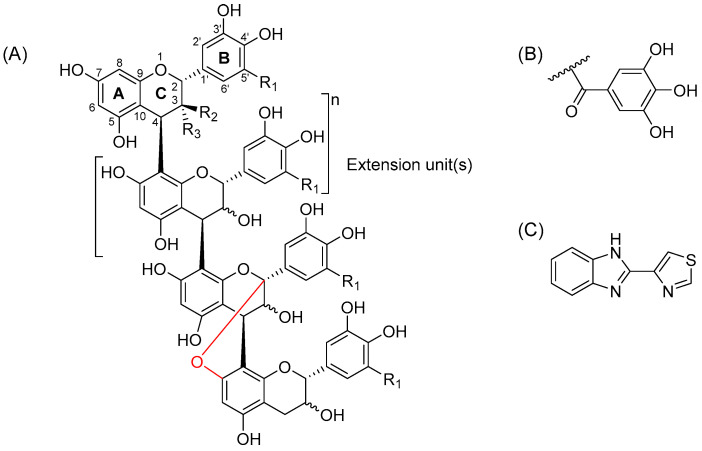
(**A**) An example of a proanthocyanidin oligomer consisting of monomeric flavan-3-ol units; (+)-catechin: R_1_ = H, R_2_ = OH, R_3_ = H; (−)-epicatechin: R_1_ = H, R_2_ = H, R_3_ = OH; (+)-gallocatechin: R_1_ = OH, R_2_ = OH R_3_ = H; and (−)-epigallocatechin: R_1_ = OH, R_2_ = H, R_3_ = OH. An A-type bond between the C2 of the C-ring and O7 of the A-ring is highlighted in red (C2 → O → C7 linkage). The ether bond can also be a C2 → O → C5 linkage. (**B**) Galloyl group, which, along with other substituents, can be attached to the O3 position in the C-ring of the monomeric unit. (**C**) The anthelmintic used in the study, thiabendazole.

Plant proanthocyanidins (PAs, syn. condensed tannins) are a substantial sub-group of specialized plant metabolites. PAs are oligomers or polymers consisting of flavan-3-ol monomeric units. The most common monomers of which these higher degree chains are formed are (epi)catechin and (epi)gallocatechin units, which are also referred to as procyanidins (PC) and prodelphinidins (PD), respectively. The monomeric units forming a PA oligomer are typically attached to each other by C4 → C8 or C4 → C6 linkages, but the structures can also contain one or more additional ether bonds from C2 to either C5 or C7 (Figure 1). PAs with these additional ether bonds are referred to as A-type PAs, while PAs containing only the carbon–carbon bonds are called B-type. These highly diverse oligomers and polymers can also have other substituents attached to their monomeric units, e.g., galloyl or glycyl groups (Figure 1).

In plants, PAs seldom appear as single oligomers or polymers but rather as mixtures of compounds of different sizes, making them elute as a chromatographic hump in reversed-phase liquid chromatography (LC). Because of their characteristic elution properties, the purification of pure PA polymers has proven difficult, while individual oligomers up to pentamers have been successfully purified [1,2,3,4]. However, Leppä et al. [5] developed a method to semipreparatively separate the hump into well-defined fractions. These PA fractions can be fully characterized by ultrahigh-resolution mass spectrometry and, thereby, the sophisticated differences in the fractions can be observed. As PAs are naturally present as complex mixtures with a great variation in their structures, their composition can also be described by their mean degree of polymerization (mDP) and by the number of PC and PD units, which is usually referred to as the PC/PD ratio or as prodelphinidin percent (PD-%).

PAs have been extensively studied for their bioactivities, especially for their protein precipitation capacity [6] and anthelmintic activity [7,8,9,10,11,12,13,14]. These bioactivities and the ubiquitous and structurally diverse nature of PAs have led them to being compounds of interest in battling the worldwide problem of anthelmintic resistance [11,15,16,17]. PAs, along with other natural compounds, could also offer a partial solution to decrease the emission of greenhouse gases arising from the rumination processes of farm animals [15,18,19,20]. The use of natural compounds is appealing for their ease of use, as they either occur naturally in some animal feed, such as in sainfoin (*Onobrychis viciifolia*), or can be easily added into feed as nutraceuticals. For example, the aforementioned sainfoin contains PD-rich PAs of medium to high mDP [21], and it has been widely used in the in vivo research focusing on the benefits of PAs in animal forage [11,15,22]. Due to widespread anthelmintic resistance, the nematode control in farm animals cannot rely just on medication anymore; instead, a combination of other control methods, such as lowering the infection pressure and utilizing anthelmintic forage or feed additives, is needed [23,24,25]. This kind of strategy could benefit from the combinatory usage of two anthelmintic substances, which could include structurally diverse natural compounds and commercial anthelmintics [26,27]. These could act complementarily to each other, as tannins have been proven to negatively affect helminths through all their life stages [13,20], while commercial anthelmintics target stages within the host. So far, PAs have been linked to both increasing and decreasing the efficacy of anthelmintic response when used orally together with the widely used anthelmintic ivermectin [28,29,30,31], accentuating the scarcity of information regarding the combinatory usage of natural compounds and commercial anthelmintics. This highlights the need for better understanding the consequences and underlying modes of action when using a combination of anthelmintic substances, whether tannins directly interact with these anthelmintics [32] or if the observed combinatory effects are a result of more complicated modes of action. These studies also highlight the necessity for deeper understanding of the nature of the possible interactions, as the tannin compositions behind these contradictorily results [28,29,30,31] are different.

We have previously studied the interactions of another substantial group of plant tannins, namely hydrolysable tannins (HTs), with a commercial anthelmintic, thiabendazole (TBZ, Figure 1) [32]. From these studies, we were able to confirm that tannins interacted with the chosen anthelmintic in a direct manner, and that this interaction was affected by the structural characteristics of HTs, namely the degree of galloylation and the overall flexibility of the HT structure. In the present study, the interactions of TBZ, a soluble representative of the benzimidazole group, were studied with another group of promising anthelmintic tannins, PAs. To begin with, we selected a variety of interesting PA structures, i.e., PC-rich, PD-rich, mixtures with intermediate levels of PC and PD units, structures with additional A-type bonds or those with only B-type linkages, and galloylated PAs. Subsequently, based on our previous research and by using a semipreparative high-performance liquid chromatography (HPLC) method [5,33], we extracted well-defined PA fractions from plant species abundant in PAs with the selected structural characteristics. The obtained PA fractions were then characterized by qualitative and quantitative ultrahigh-performance liquid chromatographic (UHPLC) tandem mass spectrometric (MS/MS) techniques. Interactions of these fractions with TBZ were studied by isothermal titration calorimetry (ITC) to investigate the presence of direct interactions also for this class of tannins and to determine which structural features of PAs influenced the observed interactions. In total, we analyzed the affinity of four individual flavan-3-ols and 15 different well-characterized PA fractions to TBZ.

## 2. Results and Discussion

### 2.1. Characterization of Proanthocyanidin Fractions

The PA fractions were characterized by two approaches—quantitatively by UHPLC-MS/MS via a multiple reaction monitoring method (MRM) [34] and qualitatively using ultrahigh-resolution UHPLC-MS/MS [33]. The quantitative data allowed us to obtain the mDP, PC/PD ratio, and relative galloyl content, while the qualitative approach was used to identify the interflavanoid A-type bonds in the structures and to examine the detailed composition of the different PA fractions. The results from the qualitative data were also used in supporting and confirming the results obtained from the quantitative data. The model PA fractions used in the ITC measurements represented different values for the mDP, PC/PD ratio, and number of galloyl groups or A-type bonds (Table 1). The ultrahigh-resolution mass spectra of the fractions reveal that while two fractions can seem similar or even the same in the light of the quantitative MRM data (mDP and PC/PD-ratio), there still can be large differences in the actual PA profiles. Smaller differences in the PA profiles were observed for PAs from the same plant source. The results in Table 1 reveal high diversity within the chromatographic PA humps, as fractions obtained from the same plant source can greatly differ by their mDP and PD-% as well as by their detailed PA composition obtained from the ultrahigh-resolution MS analysis.

**Figure 2:**
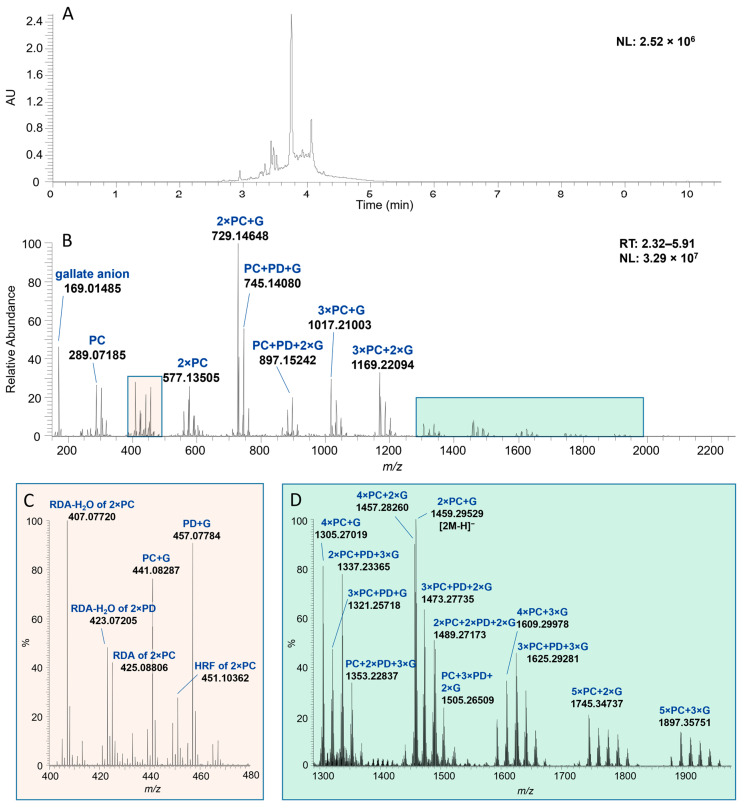
(**A**) An UV chromatogram at 280 nm and (**B**) a mass spectrum of the observed proanthocyanidin (PA) hump of the galloylated PA fraction with mDP of 5 (B-PC-G 1, Table 1) obtained from the ultrahigh-resolution MS analysis. Two *m*/*z* ranges of the mass spectrum are highlighted: (**C**) from *m*/*z* 400 to 480 and (**D**) from *m*/*z* 1290 to 1980. AU = absorbance unit, G = galloyl group, HRF = heterocyclic ring fission, NL = normalized intensity, PC = procyanidin, PD = prodelphinidin. RDA = retro-Diels–Alder, RT = retention time (min).

The order of the monomeric building blocks or the *cis*/*trans* isomeric composition could not be determined by the methods used. The detailed PA profile was characterized by examining the exact masses of the main ions from the mass spectra (Figure 2) and the characteristic fragmentation patterns for PAs; quinone–methide cleavage (QM), heterocyclic ring fission (HRF), and retro-Diels–Alder (RDA) fragmentation, which were described in more detail by Karonen et al. [33] and Sui et al. [36]. For example, a B-type oligomer consisting of purely (epi)catechin building blocks would result in a PC oligomeric series of *n* × 288 + 2, producing singly charged ions with *m*/*z* at 289, 577, 865, 1153, and 1441. A similar PD oligomeric series is typically a manifold of *n* × 304 + 2 ((epi)gallocatechins), corresponding to singly charged ions with *m*/*z* at 305, 609, 913, 1217, and 1521, for instance. As the PC and PD units differ from one another by the degree of hydroxylation, similar ions with an *m*/*z* difference of 16 Da are characteristic for PC/PD mixtures. Smaller ions, similar to those in the previously described (epi)catechin and (epi)gallocatechin series, can also be fragmentation products of higher oligomers. Higher PA oligomers form multiply charged ions when electrospray ionization is used. The efficiency of ionization decreases as the degree of polymerization of PAs increases [37], and therefore, the abundance of ions is lower for higher oligomers. For example, a series of PAs starting from heptamers can be observed as ions of *m*/*z* at 1008 + *n* × 144 or at 1064 + *n* × 152 for PC and PD series, respectively. As can be seen in B-PC-G 2 (Figure S13), low molecular weight compounds (*m*/*z* at 316 and 547) tend to have better ionization, and therefore, they seem to be more intensive in the mass spectra in comparison to PAs. However, the amount of these other phenolic compounds in this particular fraction is minor compared to the amount of PA oligomers and polymers, as also supported by the UV absorbance.

Some of the fractions used in this study contained PA oligomers with A-type bonds (Table 1). We could observe the presence and the number of these bonds, as an oligomer containing an A-type bond appears with a mass difference of 2 Da if compared to its B-type counterpart in the mass spectrum. However, the precise locations of the A-type bonds were not identified. For example, an A-type trimer consisting of three PD units and having one additional ether bond appears as a singly charged ion at *m*/*z* 911, while its B-type counterpart appears as an ion at *m*/*z* 913 (Figures S1 and S7). However, a careful investigation is required to confirm that an alleged A-type bond actually is an interflavanoid ether linkage and not an oxidation or fragmentation product of a B-type PA, which both can lead to a perceived difference of 2 Da. The retention time along with the product ion scan by MS/MS methodology can be used to distinguish A-type PAs from the oxidation or fragmentation products of B-type PAs; for example, A-type PAs tend to elute later compared to corresponding to B-type PAs [33,38]. The presence of A-type linkages can also be confirmed by the characteristic fragmentation patterns of PAs, including the HRF, RDA, and QM cleavage [33,36,39]. This kind of investigation has been conducted for all the PAs used in this study, and the A-type linkages are represented in Figures S1–S6 and Tables S1–S6 in the Supporting Information.

The galloylation of a PA can be detected by the presence of an ion at *m*/*z* 169 (corresponding to gallate anion) and by the addition of 152 Da to the observed mass for a nongalloylated PA oligomer. For an example, a PC oligomeric series with one galloyl group attached to each PA structure would exhibit singly charged ions at *m*/*z* 729 (dimer), 1017 (trimer), 1305 (tetramer), and 1593 (pentamer). The number of galloyl groups attached to a PA oligomer is not limited to only one and can be taken into account as an extra addition of 152 Da. Special care should be taken when interpreting galloylated PAs using integer mass values, as the addition of one PD unit (304 Da) equals the addition of two galloyl groups (2 × 152 Da) into an oligomer, leaving room for misinterpretation. This, however, can be avoided by the use of high-resolution MS, as the accurate masses of these two differ adequately. For example, the calculated *m*/*z* ratio for a singly charged B-type tetramer with three PC units and one PD unit would be 1169.25685, while the calculated *m*/*z* for a B-type PC trimer with two galloyl groups would be 1169.22046 (Figure 2).

In Figure 2 and Table 2, a model characterization is demonstrated for a galloylated PA fraction, B-PC-G 1. Similar series of ions can be detected in this fraction as described above at *m*/*z* 577, 729, 897, 1017, and 1169 corresponding to PAs consisting of PC units alongside with the ions with 16 Da mass difference corresponding for PAs with PD units. PAs in this specific fraction contain both PC and PD units, but as the relative abundance of PC ions is higher, this fraction is characterized as PC-rich. This also corresponds quite well with the MRM data showing that only 28% of PAs are PDs (Table 1). The ion at *m*/*z* 169 corresponds to the gallate anion, and the ion at *m*/*z* 289 corresponds either to (epi)catechin or to a fragment of a higher oligomer (Figure 2D). The characteristic fragmentation pattern for a PC dimer is shown in Figure 2C: at *m*/*z* 451 (HRF fragment of dimeric PC), 425 (RDA fragment of dimeric PC), and 407 (the sequential loss of H_2_O from the RDA fragment). In addition, ions at *m*/*z* 423 (the sequential loss of H_2_O from the RDA fragment of a PD dimer), 441 and 457 are detected, the two latter ones corresponding to galloylated (epi)catechins and (epi)gallocatechins, respectively. In Figure 2D, minor ions at the *m*/*z* range from 1290 to 1980 represent higher galloylated PC/PD oligomers characterized above. In Table 2, a detailed characterization of PAs in fraction B-PC-G 1 with their degree of polymerization, molecular formulae, calculated exact masses, linked monomeric composition, and the ions observed, is shown. By comparing the information in Table 1 with the ultrahigh-resolution MS data in Table 2, and specifically the number of galloyl groups in the PAs in B-PC-G 1 fraction, with the ultrahigh-resolution MS data in Table 2, it can be observed that the approximation of the number of galloyl groups based on MRM is close to that detected for the galloylated PA oligomers in the mass spectrum. A detailed characterization of PAs was conducted similarly to all the PA fractions used in the study (Figures S1–S14 and Tables S1–S14).

### 2.2. Isothermal Titration Calorimetry Analyses

In total, we analyzed the affinity of four flavan-3-ols and 15 different PA fractions to TBZ. The resulting observed enthalpy changes (Δ*H*_obs_) ranged from 0 kJ/mol to larger exothermic ones of approximately −30 kJ/mol. These observed enthalpy changes were then used to compare data and identify trends due to changes in the structural properties of the PA fractions. Most of the titrations yielded non-sigmoidal isotherms and could not be reliably fitted. These kinds of non-sigmoidal ITC data can be fitted, for example, if the stoichiometry is known [40], but the process of ITC data fitting involves great amounts of uncertainty, and the role of statistical error in ITC data and its effect on the determination of Δ*H* and *K_a_* is well known [41,42]. To avoid overinterpretation of the data, the discussion will focus on the comparison of enthalpy changes observed. The interaction between PAs in B-PC-G 2 and TBZ were strong enough to produce a sigmoidal binding isotherm that was fitted, and corresponding thermodynamic parameters of the interaction were obtained (see Section 2.2.6). Our previous study using HTs, with the same concentrations of tannin and TBZ as used here, yielded enthalpy changes ranging from 0 to −13 kJ/mol [32]. For perspective, the affinity of PAs to proteins, one of the most common bioactivities associated with tannins in general, has been measured to produce enthalpy changes from −10 to −100 kJ/mol [43]. While the enthalpy changes (Δ*H*) measured by ITC are generally reported as kJ per mole of injectant, the observed Δ*H* are also dependent on the concentration of the analyte present in the sample cell. Consequently, no direct comparison between ITC analyses and the observed enthalpies gained from them can be made if the concentrations used in the analyses differ.

#### 2.2.1. Interaction of Flavan-3-ols to Thiabendazole

**Figure 3:**
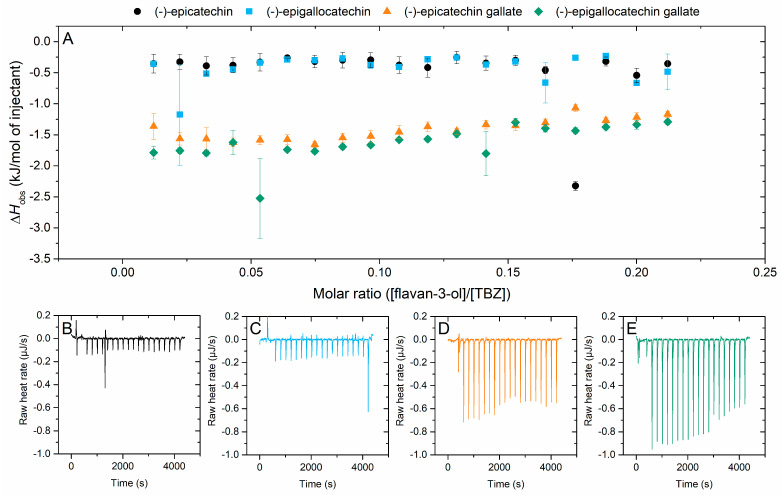
(**A**) Isotherms of titration of flavan-3-ol into thiabendazole (TBZ) after the integration of peak areas and subtraction of the control measurement (flavan-3-ol into buffer) depicting the released heat as kJ per one mole of injectant as a function of molar ratio. Obtained isothermal titration calorimetry raw data for (**B**) (−)-epicatechin, (**C**) (−)-epigallocatechin, (**D**) (−)-epicatechin gallate, and (**E**) (−)-epigallocatechin gallate.

The four flavan-3-ols included in the study were (−)-epicatechin and (−)-epigallocatechin, and their galloylated analogues, (−)-epicatechin gallate and (−)-epigallocatechin gallate. The interaction of these four monomers to TBZ yielded small and quite similar enthalpy changes: the enthalpies from the titration of epicatechin and epigallocatechin to TBZ were both nonexistent, while the interaction between the galloylated forms of the two and TBZ resulted in very small changes in enthalpy (Figure 3). From these, it can be observed that the addition of the galloyl group noticeably shifted the observed enthalpy level and had a small effect on the overall shape of the isotherm. Even though the observed enthalpies are small, and the results should thus be analyzed with caution, the favorable effect of galloylation of the flavan-3-ols on the interaction strength is visible. In previous studies, the galloylation of epicatechin and epigallocatechin has greatly increased their interaction susceptibility in comparison to their nongalloylated counterparts; for example, the galloylated flavan-3-ols have exhibited increased membrane fluidity reduction, which strengthened their antiplaque and hepatoprotective effects [44]. The presence of an additional hydroxyl group in the B ring of epigallocatechin and epigallocatechin gallate did not strengthen the interaction with TBZ as compared to the monomers with smaller degrees of hydroxylation, epicatechin and epicatechin gallate.

#### 2.2.2. Effect of Mean Degree of Polymerization on the Interaction to Thiabendazole

**Figure 4:**
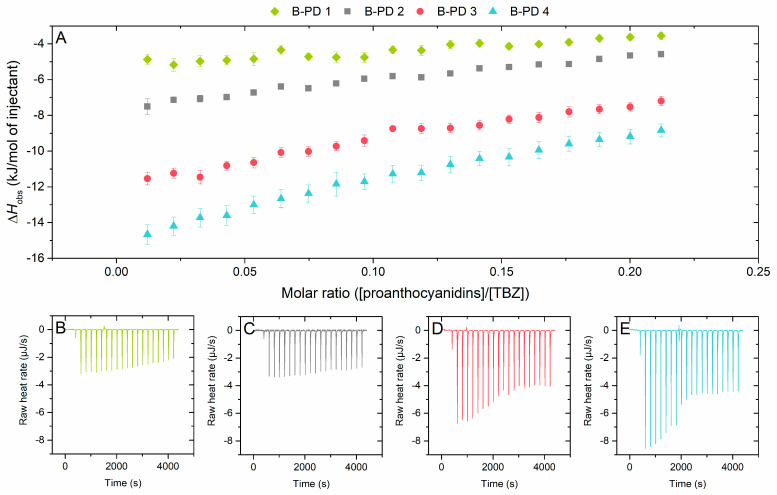
(**A**) Isotherms of titration of proanthocyanidins (PAs) into thiabendazole (TBZ) after the integration of peak areas and subtraction of the control measurement (PA into buffer) depicting the released heat as kJ per one mole of injectant as a function of molar ratio. Comparison of the effect of mDP on the observed enthalpies using different fractions of B-type PD-rich plant source: B-PD 1 (mDP 12, PD 98%), B-PD 2 (mDP 12, PD 96%), B-PD 3 (mDP 16, PD 95%), and B-PD 4 (mDP 18, PD 94%). The corresponding isothermal titration calorimetry raw data for (**B**) B-PD 1, (**C**) B-PD 2, (**D**) B-PD 3, and (**E**) B-PD 4. Individual PAs in the fractions used are shown in Figures S7–S10 and Tables S7–S10.

The effect of the mDP of PAs on their interaction with TBZ was tested by using PA fractions of different mDPs from the same plant species to minimize the variability of the overall PA composition (Figure 4). The fractions obtained from the B-PD plant source had small variability in the PD-% of their composition (range of 94–98%) but were similar enough to be compared in relation to their mDP. It should be noted that while B-PD 1 and B-PD 2 have the same integer value reported for the mDP based on the quantitative data (Table 1), the inspection of the ultrahigh-resolution mass spectra (Figures S7 and S8, Tables S7 and S8) reveals a slightly higher degree of oligomerization for B-PD 2 along with slightly larger quantities of PC units in the oligomers. Therefore, it can be observed that the increase in the mDP of PAs leads to the increase in the observed enthalpy change (Δ*H*_obs_), i.e., stronger interaction (Figure 4).

**Figure 5:**
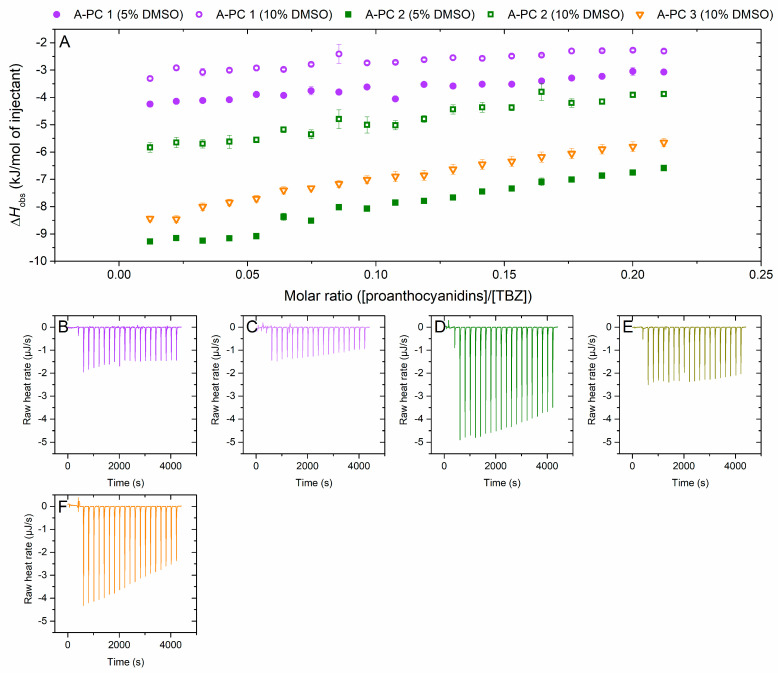
(**A**) Isotherms of titration of proanthocyanidins (PAs) into thiabendazole (TBZ) after the integration of peak areas and subtraction of the control measurement (PA into buffer) depicting the released heat as kJ per one mole of injectant as a function of molar ratio. Comparison of the effect of mDP and the used DMSO-% using fractions from the A-type PC-rich plant source: A-PC 1 (mDP 6, PD 1%), A-PC 2 (mDP 10, PD 1%), and A-PC 3 (mDP 13, PD 1%). Solid symbols represent 5% DMSO and symbols with a white center are the results obtained by using 10% DMSO. For A-PC 3, only results with 10% DMSO were obtained due to problems with solubility. The corresponding isothermal titration calorimetry raw data for (**B**) A-PC 1 with 5% DMSO, (**C**) A-PC 1 with 10% DMSO, (**D**) A-PC 2 with 5% DMSO, (**E**) A-PC 2 with 10% DMSO, and (**F**) A-PC 3 with 10% DMSO. Individual PAs in the fractions used are shown in Figures S4–S6 and Tables S4–S6.

The effect of mDP was also investigated with an A-type PC-rich series (Figure 5). This series proved to be challenging, as the fraction with the highest mDP was not soluble in 5% DMSO and had to be measured in 10% DMSO instead. This resulted in a smaller enthalpy change than expected for the A-PC 3 fraction based on the previously described mDP set (B-PD 1–4) where the enthalpy change increased along with the mean molecular size of the fractions (Figure 4), and therefore, the impact of the altered DMSO-% on the interaction was questioned. As a result, the whole A-PC series was analyzed also in 10% DMSO, confirming the hindering effect of DMSO on the interaction. The A-type linkage increases the rigidity of the PA and makes the PA more hydrophobic [45], as also proved by the solubility issues present here.

The increasing molecular size of PAs has been previously connected to their stronger interaction with proteins [46,47,48], and a similar result can be observed here for small molecule interactions as well. The higher the PAs in the fraction are, the more binding sites there are available for the TBZ to bind to, leading to higher observed enthalpy changes. Despite the numerous available binding sites provided by the higher PA oligomers, the shapes of the isotherms do not equilibrate at the end of the titration, meaning that all the available binding sites are not saturated. This appears to indicate that the number of binding sites available was always in excess of the amount of TBZ available, which is likely to mean that the interaction is non-specific or that there are multiple weakly associated binding sites or interactions available. Previously, hydrogen bonding has been considered as the driving force in specific tannin–protein interactions and π–π stacking in non-specific interactions [49,50].

#### 2.2.3. Effect of the Degree of Hydroxylation on the Interaction to Thiabendazole

**Figure 6:**
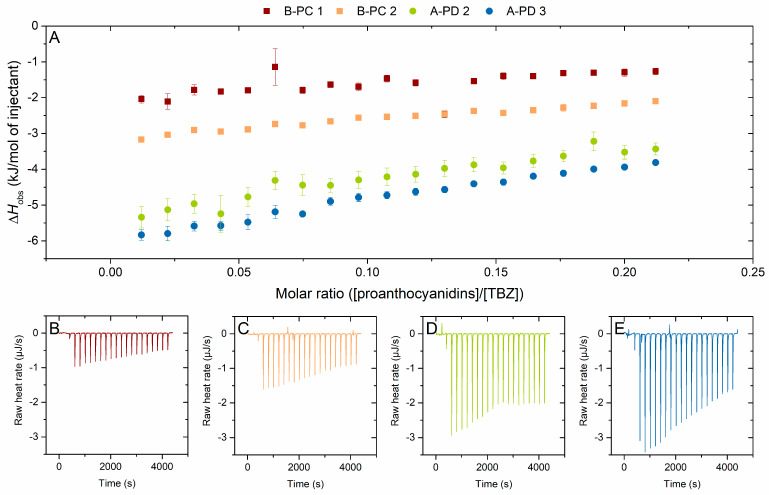
(**A**) Isotherms of titration of proanthocyanidins (PAs) into thiabendazole (TBZ) after the integration of peak areas and subtraction of the control measurement (PA into buffer) depicting the released heat as kJ per one mole of injectant as a function of molar ratio. Effect of the PC/PD ratio on the interaction strength was inspected using two pairs of fractions that were as similar to each other as possible, excluding the PC/PD ratio; (1) B-type B-PC 1 (mDP 6, PD 23%) and B-PC 2 (mDP 7, PD 5%) represented in squares, (2) A-type A-PD 2 (mDP 11, PD 93%) and A-PD 3 (mDP 11, PD 70%) represented in spheres. The corresponding isothermal titration calorimetry raw data for (**B**) B-PC 1, (**C**) B-PC 2, (**D**) A-PD 2, and (**E**) A-PD 3. Individual PAs in the fractions used are shown in Figures S2, S3, S11 and S12, and Tables S2, S3, S11 and S12.

The effect of PC/PD ratio on the interaction to TBZ was more difficult to inspect, as few fractions had similar mDP but different PC/PD ratios. However, the PC/PD ratio could be inspected with two pairs of fractions: (1) A-type PD-rich fractions with higher mDP, A-PD 2 and A-PD 3 (both with mDP of 11), and (2) B-type PC-rich fractions with smaller mDP, B-PC 1 and B-PC 2 (mDP of 6 and 7, respectively) (Figure 6). The mDP within these pairs is not exactly the same, but the variation was small enough to tentatively hypothesize the effect of PC/PD content of the PA fraction (Table 1, Figure 6). As was the case with the mDP series comparison (Figure 4 and Figure 5), where only fractions from the same plant source were compared to each other, also these comparisons were conducted using the PA fractions obtained from the very same plant species to exclude the possible additional effects caused by the slightly different PA composition. Figure 6 shows that larger enthalpy changes were observed for the fractions with higher PC-%—though it should be noted that also the mDP is slightly higher for these PC-rich fractions which might also account for the differences observed. Therefore, it can be deduced that the PC/PD ratio of PAs does not heavily influence their interaction with TBZ. Here, the PC-% changes only by 20% for both fraction pairs, and it is possible that a larger change in the PC/PD ratio in the PA structures could have shown differences.

As mentioned before, no significant differences in enthalpy changes were observed for the flavan-3-ol monomers epicatechin and epigallocatechin (Figure 3). The overall small heats from the titration to TBZ could obscure the effect of PD-% for the flavan-3-ol monomers, or, that TBZ could come close to the phenolic rings of the monomers regardless of the hydroxylation degree, whereas with higher PAs, the smaller degree of hydroxylation would affect the interaction susceptibility. For other types of tannin interactions, the higher PD share has been connected to stronger interaction as the hydroxyl groups participate in the formation of hydrogen bonds that are generally affiliated with tannin–macromolecule interactions [43]. For instance, Simon et al. (2003) have concluded that hydrogen bonding between proline carbonyls and catechol hydroxyl groups would be the driving force for the interaction [51]. Here, no corresponding effect was observed, and it is possible that the interactions are of stacking type due to aromatic π bonds. However, the enthalpy changes seen here are very small, and the effect of PD-% of PAs on their interaction with TBZ would require studies with PA fractions with larger PD-% differences, while the mDP of PAs and the linkage type between their monomeric units stay the same.

#### 2.2.4. Effect of Galloylation on the Interaction to Thiabendazole

**Figure 7:**
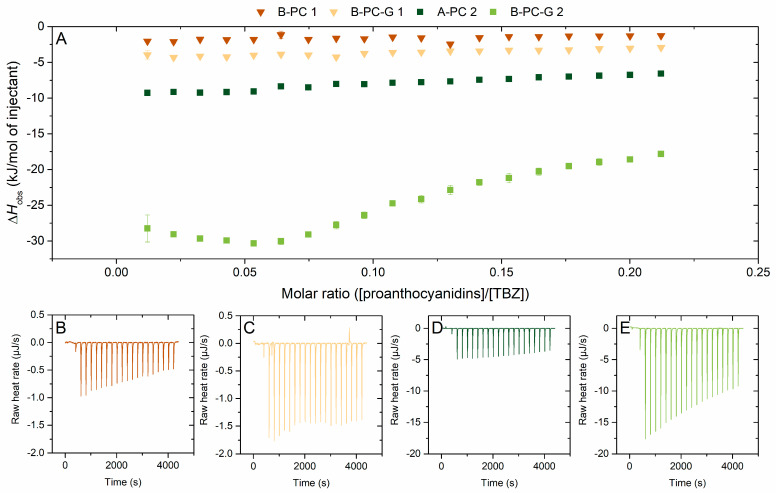
(**A**) Isotherms of titration of proanthocyanidins (PAs) into thiabendazole (TBZ) after the integration of peak areas and subtraction of the control measurement (PA into buffer) depicting the released heat as kJ per one mole of injectant as a function of molar ratio. The effect of galloyl groups in the PA structures was inspected using two pairs of fractions; (1) B-PC 1 (nongalloylated, mDP 6, PD 23%) and B-PC-G 1 (galloylated, mDP 5, PD 28%), (2) A-PC 2 (nongalloylated, mDP 10, PD 1%, A-type) and B-PC-G 2 (galloylated, mDP 10, PD 12%). The corresponding isothermal titration calorimetry raw data for (**B**) B-PC 1, (**C**) B-PC-G 1, (**D**) A-PC 2, and (**E**) B-PD-G 2. Individual PAs in the fractions used are shown in Figure 2 and Figures S5, S11 and S13, and Table 2 and Tables S5, S11 and S13. All the fractions are B-type PAs unless otherwise stated.

For understanding the effect of galloylation on the PA–TBZ interaction, PA fractions from different plant sources were used (Figure 7). For both fraction pairs (nongalloylated and galloylated) used for the comparison, the interaction of the galloylated fraction with TBZ yielded larger observed enthalpy changes, i.e., stronger interaction. For the B-PC-G 2 fraction, the strength of the interaction was high enough to yield a sigmoidal shape of the binding isotherm. This isotherm was fitted, and the obtained thermodynamic parameters are discussed further in Section 2.2.6. The galloylated fractions stand out even when the overall effect of mDP, i.e., a strongly beneficial structural parameter, was removed by examining the observed enthalpy change as kJ per monomeric unit rather than per mole of injectant (Figure S15). By examining the data this way, the galloylated PA fraction with smaller mDP, B-PC-G 1, showed significantly higher observed enthalpy changes than when comparing the enthalpies per molarity, strengthening the hypothesis of increased interaction due to the presence of galloyl groups.

The beneficial effect of galloylation on the interaction strength was expected based on our previous results utilizing HTs [32]. Galloylation also has a similar effect on other interactions involving tannins, as it increases the overall flexibility of the molecule and provides a larger amount of binding sites for really any type of molecule with a tendency to utilize hydrogen bonding and/or π–π stacking in its interaction [52,53,54]. A similar effect of galloylation has also been shown for the associations of dimeric procyanidins to proline-rich proteins, as a higher *K_a_* value was obtained for procyanidin B2 3′-*O*-gallate than for procyanidin B2 [55]. In these procyanidin–salivary protein interactions, hydrophobic interactions and hydrogen bonds have been reported to be present in all interactions, and they can play complementary roles depending on the structures of the protein and the procyanidin involved [55,56].

The extent of the increased interaction due to two to three additional galloyl groups attached to each PA is quite interesting, especially in the bigger picture. For the B-PC-G 2 fraction, the mDP is relatively high and the fraction is PC-rich which, based on the previous interpretations (Figure 4, Figure 5 and Figure 6), is a very good combination of structural parameters favoring the strong interaction with TBZ. Contradictory to studies on PA–protein interactions [45,57], here the effect of galloylation seems to override the effect of mDP, as our dataset also contains fractions with mDP as high as 18, which is almost double compared to the B-PC-G 2 fraction with the largest observed enthalpy change. This result is in accordance with our previous study using HTs, as also there, the strongest interaction was not found with the highest polymerization degree but with the highest number of galloyl groups. In addition to degree of galloylation, also the order and isomerization of the adjacent monomeric units and the placement of the galloyl groups could play a role in the strong interaction of this particular fraction to TBZ, but our data do not reveal these characteristics. According to our results, the most favorable effect is reached with a slightly higher value for mDP, which is revealed by the comparison of the Δ*H*_obs_ for B-PC-G 1 and B-PC-G 2, corresponding to mDPs of 5 and 10, respectively, to the Δ*H*_obs_ for the nongalloylated A-PC 2 with an mDP of 10 (Table 1). The fraction B-PC-G 1 exhibits a significantly smaller enthalpy change compared to A-PC 2, which is contradictory to our previous interpretation of the effect of galloylation exceeding the effect of mDP. This would indicate that even in the presence of galloyl groups, a higher mDP is required for stronger binding to TBZ. The degree of galloylation for the galloylated fractions also differs, 2.1 for B-PC-G 1 (Table 1) and 3.6 for B-PC-G 2 (Table 1), which may contribute to the magnitude of observed differences as well.

#### 2.2.5. Interaction of A-Type Proanthocyanidins to Thiabendazole

**Figure 8:**
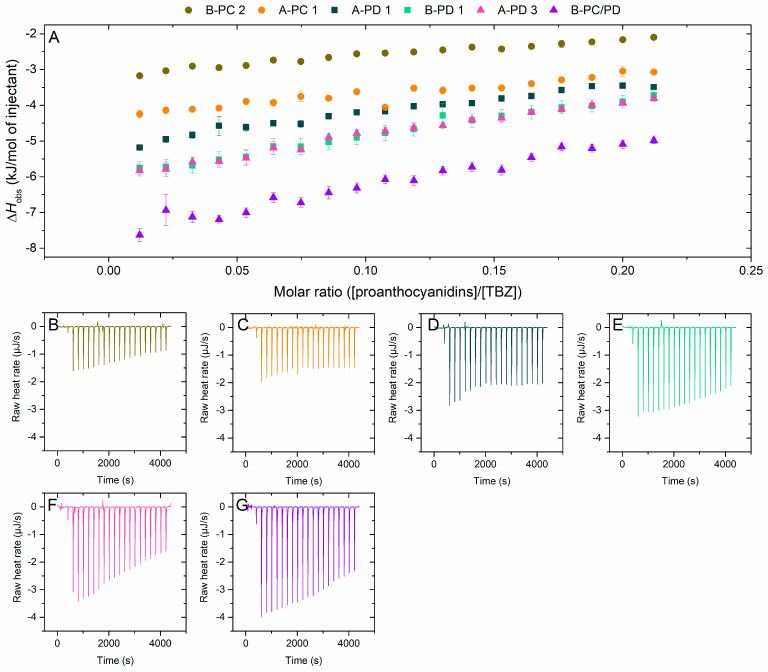
(**A**) Isotherms of titration of proanthocyanidins (PAs) into thiabendazole (TBZ) after the integration of peak areas and subtraction of the control measurement (PA into buffer) depicting the released heat as kJ per one mole of injectant as a function of molar ratio. Comparison of the effect of additional ether linkages in the structure, i.e., A-type bonds, on the interaction to TBZ was conducted using three pairs of fractions, each pair marked with the same symbols (sphere, square, and triangle); (1) A-PC 1 (A-type, mDP 6, PD 1%) and B-PC 2 (B-type, mDP 7, PD 5%), (2) A-PD 1 (A-type, mDP 11, PD 95%) and B-PD 1 (B-type, mDP 12, PD 98%), and (3) A-PD 3 (A-type, mDP 11, PD 70%) and B-PC/PD (B-type, mDP 11, PD 69%). The corresponding isothermal titration calorimetry raw data for (**B**) B-PC 2, (**C**) A-PC 1, (**D**) A-PD 1, (**E**) B-PD 1, (**F**) A-PD 3, and (**G**) B-PC/PD. Individual PAs in the fractions used are shown in Figures S1, S3, S4, S7, S12 and S14, and Tables S1, S3, S4, S7, S12 and S14.

The effect of the additional ether linkages between the monomeric units, i.e., the A-type bonds, was also evaluated (Figure 8). The results indicate that A-type linkages do not affect the interaction to TBZ, as the observed enthalpy changes obtained for the fractions do not show a consistent trend and as the differences between the compared enthalpy changes are small. For example, when comparing A-PC 1 and B-PC 2, we can observe that the A-type PAs have stronger interactions with TBZ (Figure 8). The mass spectra of these fractions are similar (Figures S4 and S12), although the A-type A-PC 1 exhibits more intensive ions at *m*/*z* values corresponding to dimers and to oligomers ranging from heptamers to undecamers. These differences, however, are slight, and thus, the difference in interaction enthalpies is more likely to be caused by the multiple A-type bonds in the A-PC 1 fraction. The opposite effect, however, is observed for the fractions A-PD 3 and B-PC/PD, where the B-type PAs exhibited larger enthalpy changes in their interaction with TBZ. The inconsistency in these comparisons is interesting, as it prompts more inspection of the other possible structural characteristics that could influence the interaction. When inspecting the latter pair, the A-type A-PD 3 fraction has both A- and B-type oligomers, with the B-type PAs being generally more prominent (Figure S3). Thus, the difference in abundance of A-type linkages between these two fractions is smaller compared to the pair of A-PC 1 and B-PC 2. The observed result could also stem from the distribution of oligomers, even though the mDP of these two fractions is 11. For the A-type fraction (A-PD 3), the oligomeric distribution is more limited, with the tetramer exhibiting the most intensive signal (Figure S3), while the B-type B-PC/PD has a more distributed oligomeric composition and the *m*/*z* signals for pentamers to nonamers are more intensive compared to the A-type counterpart (Figure S14). A similar trend can be observed for the third fraction pair, A-PD 1 and B-PD 1, where a slightly larger enthalpy change was obtained for the B-type fraction with a more even distribution of oligomers according to the mass spectra (Figures S1 and S7). As established above (Figure 4 and Figure 5), the increase in polymerization degree also increases the interaction strength, and this could partly explain the contradictory results observed here.

#### 2.2.6. Fitting of the Sigmoidal Binding Isotherm

**Figure 9:**
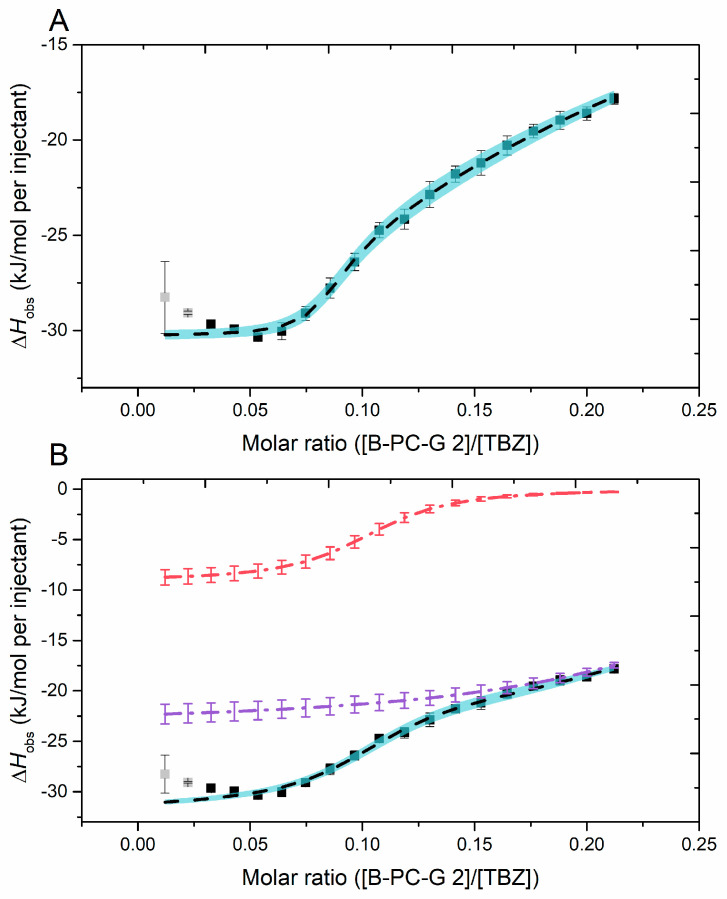
(**A**) Multiple sites model and (**B**) two independent sites model fitted to the binding isotherm obtained for the interaction between all proanthocyanidins in the B-PC-G 2 fraction (Figure S13 and Table S13) and thiabendazole (TBZ) (■). Model sums (black, dashed line) and their standard errors (blue shade) are shown for both fitting models, and for the two independent sites model (**B**), both independent fits are shown (dash–dot line): independent 1 (red) and independent 2 (purple). Data points marked in gray were not included in the fitting process. *n* = 3 for all calculated standard errors.

One fraction, galloylated and with high mDP and high PC-%, produced a sigmoidal binding isotherm under the experimental parameters of this study (B-PC-G 2, Table 1 and Figure 7). This isotherm was fitted with two different two-site binding models; by a multiple binding sites model (Figure 9A) and by a model with two individual independent binding sites (Figure 9B), the latter producing the most accurate fit (Figure 9B and Table 3). The data were also fitted by a single independent binding sites model (a single-site model), but the model did not effectively fit to the binding isotherm. In the binding isotherm (Figure 9, shown as points), the observed exothermic response (Δ*H*) increased in the first stages of the titration, which indicated cooperative binding taking place from the addition of more of the galloylated PAs [54,58]. Cooperative binding has been detected, for example, in studies on tannin–protein interactions where the tannins injected have cooperatively associated with protein-bound tannins in the sample cell by hydrophobic interactions instead of binding to different sites on the proteins and thereby affecting the stoichiometry of the interaction [56,58]. These data points were not included in the fitting process (marked in gray in Figure 9). While both of the two-site models fit well to the binding isotherm, the model with two independent binding sites (Figure 9B) exhibited smaller standard errors between replicates and more accurate Δ*H* values compared to the multiple sites model (Table 3) and is thus discussed further.

The binding model with two independent binding sites (Figure 9B) yielded three fit graphs in total: independent 1 and 2 (Figure 9B, red and purple), and a sum graph of these two graphs (Figure 9B, black). Interestingly, the independent 2 from this model was very similar compared to the rest of the data that did not produce sigmoidal isotherms, as it was slightly upwards advancing, and the observed enthalpy change did not reach zero at the end of the titration. This would indicate that the binding sites are not fully saturated at the end of the titration, which is an observation made also in our previous study [32]. This could be caused by an increasing number of available binding sites caused by the addition of more PA into the tannin–anthelmintic mixture or by some other more complex reactions involving both the tannin and the anthelmintic. The other part of the model (independent 1, Figure 9B), the sigmoidal shape, could stem from the increased interaction susceptibility caused by the combination of the high degree of galloylation and high mDP and PC/PD ratio of the fraction. Here, we investigated the interactions between relatively small compounds with equivalent molar concentrations, leading to the obtained thermodynamic parameters being somewhat different from the parameters obtained from the more traditional ITC measurements, for example from tannin into protein titrations [59] or from TBZ into macromolecule titrations [60,61]. The obtained affinity constant values, *K_a_* (M^−1^), from the model with two independent binding sites were on a similar level with the values obtained for PA–protein interactions [43] or for interactions between TBZ and human serum albumin [60] (Table 3). Notably, both of the *K_a_* values presented here are quite similar (*K_a_*_1_ of 9.9 × 10^4^ M^−1^ and *K_a_*_2_ of 3.17 × 10^4^ M^−1^, Table 3), indicating that both of the independent interactions taking place are of similar strength. A probable explanation for this could be that as the interactions progress, the PA chain undergoes conformational changes, revealing more binding sites of almost similar strength for the small TBZ to bind to.

In Table 3, the number of binding sites (*n*) is reported as the ratio of tannin to TBZ, so here, the *n*_1_ of 0.1 for the two independent sites model represents that approximately ten TBZ molecules are bound to one PA oligomer and three TBZ molecules are bound to one PA oligomer for *n*_2_. This correlates well with the observed *K_a_* values; in the first phase of the interaction, represented by *K_a_*_1_ and *n*_1_, most of the available TBZ molecules in the solution bind to the injected PA, while in the second phase, represented by *K_a_*_2_ and *n*_2_, the PA chain reveals more binding sites for TBZ, as described above. The observed enthalpy change, Δ*H*_obs_, calculated from both of the fitting models is intriguing, as typically larger enthalpy changes are recorded for the interactions with larger *K_a_* and with more ligand bound to the “macromolecule”, in this case, the *n*_1_. To confirm the results obtained from this data fitting, the obtained binding isotherm would have to reach its saturation point, which would require substantial (and solubility-wise unreachable) changes in the molarities of the used compounds.

In our previous study, we used HTs and TBZ in the same molarities as in this continuation, and thus, the results can be directly compared to each other. Although we could not reliably fit any of the HTs, we did perform initial thermodynamic analysis with the HT exhibiting the strongest interaction to TBZ, rugosin D, which contains five galloyl groups but is a smaller molecule (MW 1875.3 Da) than the B-PC-G 2 fraction (mean MW 3593.1 Da). The thermodynamic parameters obtained from this initial fitting with one independent binding site model are on a similar level to the ones obtained in this study. For example, the *n* for both of the interactions is significantly less than one, corresponding to the theory of TBZ being the ligand in this interaction; i.e., *n*_1_ of 0.1 as reported in Table 3 would correspond to ten TBZ molecules attached to one PA oligomer. As the mDP of this PA fraction is about 10, based on the quantitative and qualitative analysis, this would correspond to approximately one TBZ molecule per one monomeric unit of the PA. However, as the dataset also contained fractions with even higher mDPs, we could deduce that attached galloyl groups in the PA oligomers and polymers in B-PC-G 2 greatly increased the amount of bound TBZ. This would be consistent with the previous results, as a similar trend was also observed for HT–TBZ interaction: molecular size is a significant factor, but in the context of interactions between two smaller compounds, the role of attached functional groups takes precedence. Similar observations have been obtained for the interactions between highly polymerized (mDP = 26) and highly galloylated (72%) persimmon PAs, epigallocatechin gallate dimers and epicatechin gallate and biological membranes by Zhu et al. (2019) [62]. In their study, it was also noticed that as PAs (and also synthetic liposomes in their case) are mixtures, the resulting ITC binding isotherm is atypical in comparison to those obtained from pure ligand–receptor reactions. As a consequence, they concluded that the binding constants calculated might not be robust enough to describe the affinity of PAs to the membrane but could be quantitatively used to compare differences between compounds [62], similarly as established here for those interactions for which data could not be reliably fitted.

## 3. Materials and Methods

### 3.1. Reagents and Sample Collection

The reagents used for the ITC experiments were thiabendazole (CAS 148-79-8, PESTANAL^®^, analytical standard, ≥98.0%), dimethyl sulfoxide (DMSO, CAS 67-68-5, ≥99.9%), and buffer reagents sodium citrate dibasic dihydrate (CAS 6132-04-3, ≥99.0%) and citric acid monohydrate (CAS 5949-29-1, ≥99.0%). These reagents were purchased from Sigma-Aldrich International GmbH, St. Louis, MO, USA. All water used was type I ultrapure water prepared with a Merck Millipore Synergy UV system (Merck KGaA, Darmstadt, Germany).

The flavan-3-ols were purchased from ExtraSynthese, Genay, France, and were of the following purities: (−)-epicatechin (≥99%), (−)-epigallocatechin (≥98%), (−)-epicatechin gallate (≥97.5%), and (−)-epigallocatechin gallate (≥98%). The PA fractions were extracted from the plant materials listed in Table 1. Leaves of the plant species used were collected from the Botanical Garden of the University of Turku during spring and summer 2021, excluding *Ribes alpinum* and *Pellaea rotundifolia* leaves, which were collected from the Finnish nature during summer 2022 and from commercially purchased plants, respectively. After collection, plant materials were kept in ice to prevent the activation of natural enzymes and were frozen and lyophilized as soon as possible.

### 3.2. Plant Extraction and Fractionation

After lyophilization, the plant materials were ground to fine powder and extracted multiple times with acetone/water solution (80/20, *v*/*v*). The extraction was followed via UPLC-DAD to ensure that all PAs were extracted from the leaf material. The obtained multiple extracts from each plant were then combined, acetone was evaporated, and the aqueous extracts were lyophilized.

Sephadex LH-20 column chromatography was used to separate the PAs from other natural compounds in the extracts similarly to Leppä et al. [5]. Shortly, approximately 5–10 g of dry extract, depending on the yield of the extraction, was dissolved into 30–50 mL of water, centrifuged, and filtered through 0.45 µm polytetrafluoroethylene (PTFE) filters. The aqueous extract was then applied on a Sephadex LH-20 column (40 × 4.8 cm i.d., Kimble-Chase Kontes™ Chromaflex™, Vineland, NJ, USA). The flow rate was 5 mL/min, and the elution profile was as follows: two water fractions of 150 mL and 350 mL (Sep1a and Sep1b), 500–600 mL of methanol/water (30/70, *v*/*v*, Sep2), 500–600 mL of methanol/water (50/50, *v*/*v*, Sep3), 500–600 mL of acetone/water (30/70, *v*/*v*, Sep4), 500–600 mL of acetone/water (50/50, *v*/*v*, Sep5), and 500–800 mL of acetone/water (80/20, *v*/*v*, Sep6). The PAs eluted in the Sep6 fraction, and their yields ranged from 600 to 900 mg, depending on the plant species and the original amount of plant extract. If there was enough raw extract, the process was repeated multiple times to achieve a sufficient amount of the Sep6 fraction for the semipreparative fractionation.

Further fractionation was conducted via a semipreparative HPLC system consisting of an HPLC pump, a diode array detector, and a fraction collector. The column used was a C-18 HPLC column (Gemini^®^, 110 Å, AXIA^TM^ Packed, 150 × 21.2 mm, 10 µm, Phenomenex Inc., Torrance, CA, USA). Acetonitrile (A) and 0.1% formic acid in water (*v*/*v*) (B) were used as eluents. As previously reported [5], the flow rate was 12 mL/min, and the elution profile was the following: 0–4 min, 8% of A (isocratic gradient); 4–32 min, 8 → 55% of A (linear gradient); 32–35 min, 55 → 80% of A (linear); 35–80 min, 80 → 8% of A (column wash and stabilization). Sample preparation was performed by first diluting 125–150 mg of the PA-rich Sep6 fraction to a few drops of ethanol and then adding 2 mL of water. The dissolved sample was then centrifuged and filtered through a 0.2 µm PTFE filter. The PAs were collected into either 2 mL microcentrifuge tubes or 10 mL glass test tubes. The smaller fractions of 2 mL or 8 mL were then combined based on the UV chromatogram at 280 nm: the characteristic PA hump was first divided into six sections of equal size, resulting in six semipreparative fractions for each PA source.

### 3.3. UPLC-MS/MS Analyses

The PA composition of the obtained PA fractions was analyzed via an MRM method [34,63] and via ultrahigh-resolution MS [5,33]. The system for the MRM analyses was an Acquity UPLC system (Waters Corp., Milford, MA, USA) connected to a Xevo TQ triple-quadrupole mass spectrometer (Waters Corp., Milford, MA, USA). For the ultrahigh-resolution MS analyses, a similar UPLC system was connected to a Q Exactive Orbitrap^TM^ (Thermo Fisher Scientific GmbH, Bremen, Germany).

The column used for both instruments was an Acquity UPLC BEH Phenyl column (2.1 × 100 mm, 1.7 µmm Waters Corp., Wexford, Ireland). The column temperature was 40 °C. LC-MS grade acetonitrile (A) and 0.1% formic acid in water (*v*/*v*) (B) were used as eluents. The flow rate was 0.5 mL/min. The elution profile for all UPLC-MS/MS analyses was the same: 0–0.5 min, 0.1% of A (isocratic gradient); 0.5–5.0 min, 0.1 → 30% of A (linear gradient); 5.0–7.0 min, 30 → 40% of A (linear); 7.0–7.1 min, 40 → 90% of A (linear); 7.1–10.2 min, 90 → 0.1% of A (column wash and stabilization). Before the analysis, PA fractions were dissolved into 10% aqueous ethanol and filtered through 0.2 µm PTFE filters.

For the MRM method, a negative ionization mode was used, and heated electrospray ionization (HESI) parameters were as follows: capillary voltage 1.8 kV, desolvation temperature 650 °C, source temperature 150 °C, desolvation and cone gas (N_2_) flow rates of 1000 and 60 L/h, respectively. The MS method consisted of a full scan analysis with a mass range of *m*/*z* 150–1200 and group-specific MRM methods as previously described [34,63]. The stability of the instrument conditions was monitored throughout the analysis via external 1 µg/mL catechin standards [21,34]. Acquired data were smoothed (window size 5 scans × 2 smoothing iterations) and integrated with TargetLynx software (MassLynx V4.2 SCN982 © 2017 Waters Corp., Milford, MA, USA). Integrated PC and PD traces were converted into quantitative data by using calibration curves made separately for PC, PD, and mDP [34]. The known PC, PD, and mDP content for the calibration curves were initially obtained through thiolysis [34,64].

A negative ionization mode was also used for the collection of the ultrahigh-resolution MS data with the following HESI parameters: a spray voltage of −3.0 kV, sheath gas and auxiliary gas (N_2_) flow rates of 60 and 20, respectively, sweep gas flow rate of 0, a capillary temperature of +380 °C, and in-source collision-induced dissociation (CID) of 30 eV. For full scan MS, the mass range was *m*/*z* 150–2250 with resolution of 35 000 and automatic gain control of 3 × 10^6^. For the MS/MS analyses, a TopN method was used with the stepped normalized collision energies of 20, 50, and 80 eV, the resolution 17,500, and the automatic gain control 1 × 10^5^. Prior to analysis, the Orbitrap^TM^ was calibrated using Pierce ESI Negative Ion Calibration Solution (Thermo Fisher Scientific Inc., Waltham, MA, USA). The data were processed using Thermo Xcalibur Qual Browser software (Version 4.1.31.9, Thermo Fisher Scientific Inc., Waltham, MA, USA).

### 3.4. Isothermal Titration Calorimetry Analyses

A MicroCal iTC200 (Malvern Panalytical Ltd., Malvern, UK) was used for the experiments. Water-into-water titrations and an EDTA test kit provided by the manufacturer were used to monitor the state of the instrument throughout the data acquisition period. The experiment protocol was similar to the one used in our previous study [32]. Shortly, the concentrations of both the tannin and the TBZ solution were 3 mM, and pH 3.6 citrate buffer with 5% DMSO was used. Fraction A-PC 3 had to be diluted in 10% DMSO due to solubility issues. The other PA fractions from the same plant source, A-PC 1 and A-PC 2, were measured in both 5% and 10% DMSO. Weighed TBZ was first dissolved into DMSO, and this stock solution was diluted with the buffer to the wanted TBZ and DMSO concentrations. The experiment temperature was 40 °C, the injection volume of the first injection was 0.4 µL, and then it was 2 µL for the following 19 injections. Reference power (RP) was 5 µCal/s, stirring 750 rpm, initial delay before the first injection 400 s, and spacing 200 s between injections. Experiments consisted of one control titration of tannin into buffer and three replicates of titration of PA into TBZ. Additional control experiments of buffer into buffer and buffer into TBZ were also conducted, but these resulted in small enthalpy changes of equal size and were not taken into account during data processing. The obtained raw data were processed using NanoAnalyze software (v. 3.12.0, 2008, TA Instruments, New Castle, DE, USA). After processing, isotherms depicting the released heat (kJ) per mole of injectant as a function of molar ratio of PA to TBZ were obtained. Each of the measured three replicates were processed individually, and standard errors between the replicates were calculated. The sigmoidal shaped isotherm produced by a B-PC-G 2 fraction was fitted with a model for multiple binding sites and two separate independent binding sites with the same NanoAnalyze software (v. 3.12.0, 2008, TA Instruments).

## 4. Conclusions

The affinities of four flavan-3-ols and 15 well-defined PA fractions to a commercial anthelmintic drug, a benzimidazole, TBZ, were analyzed via ITC. The results indicate a strong correlation of the presence of galloyl groups and higher mDP of PAs to increased interaction strength. The effect of galloylation was predominant in the high mDP scale; a galloylated fraction with an mDP of 10 produced a twofold interaction strength compared to a fraction with an mDP of 18. Other well-known structural characteristics of PAs were also evaluated; PC-rich fractions seemed to have slightly stronger interactions with TBZ as compared to PD-rich fractions, while the presence of A-type linkages in the PA structures had minimal impact on the interaction. Compared to our previous study, the PAs tended to have higher affinities to the anthelmintic than the HTs of similar molarities.

In the future, similar studies should be conducted with a larger variability of different commercial anthelmintics, such as examples from the macrocyclic lactones and other benzimidazoles, as the TBZ used in this study only acts as a representative of the benzimidazole group. More information on the binding sites could be obtained via studies using NMR and molecular docking if possible. In vitro and in vivo studies should be conducted in the future with known tannin compositions to study whether the interactions observed in our study take place also in the presence of other targets of tannins, such as proteins, and later to explore the combined effect of tannins and anthelmintics on living worms. Another interesting field of study would be the pharmacokinetic and/or pharmacodynamic properties of tannin–anthelmintic complexes which could elucidate the impact of complex formation on the overall anthelmintic efficacy.

## Figures and Tables

**Table 1:** The plant origin and structural characteristics of the proanthocyanidin (PA) fractions used. The number of galloyl groups and A-type bonds for each fraction are presented as approximations per one oligomer or polymer. The number of galloyl groups is relative to the galloyl content calculated from the quantitative data and is in accordance with the qualitative ultrahigh-resolution MS data. mDP = mean degree of polymerization, PD-% = prodephinidin-%, mDa = mean molecular weight.

Scientific Name	Common Name	Code	mDP	PD-%	#Galloyls	#A-Type Bonds ^a^	mDa ^b^
*Pellaea* *rotundifolia*	Button fern	A-PD 1	11	95%	0.0	1.0	3336.6
		A-PD 2 ^c^	11	93%	0.0	0.8	3332.6
		A-PD 3	11	70%	0.0	0.5	3323.0
*Ixora coccinea*	Jungle geranium	A-PC 1	6	1%	0.0	2.0	1727.2
		A-PC 2 ^c^	10	1%	0.0	3.0	2993.1
		A-PC 3	13	1%	0.0	3.0	3742.7
*Ribes alpinum*	Alpine currant	B-PD 1	12	98%	0.0	0.0	3540.8
		B-PD 2	12	96%	0.0	0.0	3700.7
		B-PD 3	16	95%	0.0	0.0	4900.4
		B-PD 4	18	94%	0.0	0.0	5322.0
*Cephalotaxus harringtonia* *drupacea*	Japanese plum yew	B-PC 1 ^c^	6	23%	0.0	0.0	1810.5
		B-PC 2	7	5%	0.0	0.0	1879.5
*Coccoloba* *uvifera*	Sea grape	B-PC-G 1 ^c^	5	28%	2.1	0.0	1663.1
		B-PC-G 2	10	12%	3.6	0.0	3593.1
*Podocarpus* *macrophyllus*	Yew plum pine	B-PC/PD	11	69%	0.0	0.0	3174.8

^a^ Approximation based on the ultrahigh-resolution MS data. The number of A-type bonds can be smaller than one, as not all PA oligomers in the fraction contain these additional ether bonds. ^b^ Calculated from the quantitative MS/MS data (mDP and PC/PD ratio) with the number of A-type bonds and possible attached galloyl groups taken into account. ^c^ These PA fractions have been previously reported by Suominen et al. [35].

**Table 2:** Characterization of the main ions of the galloylated proanthocyanidin (PA) fraction with mDP of 5 (B-PC-G 1, Table 1) obtained from the ultrahigh-resolution MS analysis: the degree of polymerization (DP), molecular formulae, calculated exact mass, the monomeric composition of the detected compounds and the molecular ion observed. PC = procyanidin, PD = prodelphinidin, and G = galloyl group.

DP	Molecular Formula	Calculated Mass	Monomeric Composition	[M-H]^−^	[2M-H]^−^
-	C_7_H_6_O_5_	170.02153	gallate anion	169.01485	
1	C_15_H_14_O_6_	290.07904	PC	289.07185	
2	C_30_H_26_O_12_	578.14243	2 × PC	577.13505	
2	C_37_H_30_O_16_	730.15339	2 × PC + G	729.14648	
2	C_37_H_30_O_16_	730.15339	2 × PC + G		1459.29529
2	C_37_H_30_O_17_	746.14831	PC + PD + G	745.14080	
2	C_37_H_30_O_18_	762.14322	2 × PD + G	761.13542	
2	C_44_H_34_O_20_	882.16435	2 × PC + 2 × G	881.15817	
2	C_44_H_34_O_21_	898.15927	PC + PD + 2 × G	897.15242	
2	C_44_H_34_O_22_	914.15418	2 × PD + 2 × G	913.14639	
3	C_52_H_42_O_22_	1018.21678	3 × PC + G	1017.21003	
3	C_52_H_42_O_23_	1034.21170	2 × PC + PD + G	1033.20370	
3	C_52_H_42_O_24_	1050.20661	PC + 2 × PD + G	1049.19849	
3	C_59_H_46_O_26_	1170.22774	3 × PC + 2 × G	1169.22094	
3	C_59_H_46_O_27_	1186.22266	2 × PC + PD + 2 × G	1185.21531	
3	C_59_H_46_O_28_	1202.21757	PC + 2 × PD + 2 × G	1201.20948	
4	C_67_H_54_O_28_	1306.28017	4 × PC + G	1305.27019	
4	C_74_H_58_O_32_	1458.29113	4 × PC + 2 × G	1457.28260	
4	C_74_H_58_O_33_	1474.28605	3 × PC + PD + 2 × G	1473.27735	
5	C_82_H_66_O_34_	1594.34356	5 × PC + G	1593.33421	
4	C_81_H_62_O_36_	1610.30209	4 × PC + 3 × G	1609.29978	
5	C_89_H_70_O_38_	1746.35452	5 × PC + 2 × G	1745.34737	
5	C_89_H_70_O_39_	1762.34944	4 × PC + PD + 2 × G	1761.33710	
5	C_96_H_74_O_42_	1898.36548	5 × PC + 3 × G	1897.35751	

**Table 3:** Estimated thermodynamic binding parameters obtained by multiple binding sites model and two independent binding sites model used to fit the sigmoidal binding isotherm obtained for the interaction of B-PC-G 2 fraction to thiabendazole (TBZ).

Model	*Ka*_1_ (M^−1^) ^a^	*Ka*_2_ (M^−1^) ^a^	*n*_1_ ^b^	*n*_2_ ^b^	Δ*H*_1_ (kJ/mol) ^c^	Δ*H*_2_ (kJ/mol) ^c^
Multiple Sites	(5.99 ± 2.08) × 10^5^	(4.66 ± 0.84) × 10^2^	0.08 ± 0.05	0.16 ± 0.02	−30 ± 0	−165 ± 24
Two Independent Sites	(9.99 ± 0.83) × 10^4^	(3.17 ± 0.55) × 10^4^	0.10 ± 0.00	0.30 ± 0.01	−9 ± 1	−23 ± 1

^a^ *K_a_*_1_ and *K_a_*_2_ are the affinity constants (syn. equilibrium binding constant) for the two sets of binding sites. ^b^ *n*_1_ and *n*_2_ represent the corresponding binding sites for the compound in the sample cell. ^c^ Δ*H*_1_ and Δ*H*_2_ represent the corresponding enthalpy changes.

## Data Availability

The data presented in this study are available on request from the corresponding author.

## Supplementary Materials

The following supporting information can be downloaded at https://www.mdpi.com/article/10.3390/molecules29153492/s1, Figure S1: An UV chromatogram at 280 nm, total ion chromatogram (TIC) and a mass spectrum of the observed proanthocyanidin (PA) hump of the PA fraction A-PD 1 obtained from the ultrahigh-resolution MS analysis. AU = absorbance unit, G = galloyl group, NL = normalized intensity, PC = procyanidin, PD = prodelphinidin, RT = retention time (min), Table S1: Characterization of the main ions of the proanthocyanidin (PA) fraction A-PD 1 obtained from the ultrahigh-resolution MS analysis: the degree of polymerization (DP), molecular formulae, calculated exact mass, the monomeric composition of the PA oligomer and the molecular ion observed. PC = procyanidin, PD = prodelphinidin, and RDA = retro-Diels–Alder, Figure S2: An UV chromatogram at 280 nm, total ion chromatogram (TIC) and a mass spectrum of the observed proanthocyanidin (PA) hump of the PA fraction A-PD 2 obtained from the ultrahigh-resolution MS analysis. AU = absorbance unit, G = galloyl group, NL = normalized intensity, PC = procyanidin, PD = prodelphinidin, RT = retention time (min), Table S2: Characterization of the main ions of the proanthocyanidin (PA) fraction A-PD 2 obtained from the ultrahigh-resolution MS analysis: the degree of polymerization (DP), molecular formulae, calculated exact mass, the monomeric composition of the PA oligomer and the molecular ion observed. PC = procyanidin and PD = prodelphinidin, Figure S3: An UV chromatogram at 280 nm, total ion chromatogram (TIC) and a mass spectrum of the observed proanthocyanidin (PA) hump of the PA fraction A-PD 3 obtained from the ultrahigh-resolution MS analysis. AU = absorbance unit, G = galloyl group, NL = normalized intensity, PC = procyanidin, PD = prodelphinidin, RT = retention time (min), Table S3: Characterization of the main ions of the proanthocyanidin (PA) fraction A-PD 3 obtained from the ultrahigh-resolution MS analysis: the degree of polymerization (DP), molecular formulae, calculated exact mass, the monomeric composition of the PA oligomer and the molecular ion observed. PC = procyanidin and PD = prodelphinidin, Figure S4: An UV chromatogram at 280 nm, total ion chromatogram (TIC) and a mass spectrum of the observed proanthocyanidin (PA) hump of the PA fraction A-PC 1 obtained from the ultrahigh-resolution MS analysis. AU = absorbance unit, G = galloyl group, NL = normalized intensity, PC = procyanidin, PD = prodelphinidin, RT = retention time (min), Table S4: Characterization of the main ions of the proanthocyanidin (PA) fraction A-PC 1 obtained from the ultrahigh-resolution MS analysis: the degree of polymerization (DP), molecular formulae, calculated exact mass, the monomeric composition of the PA oligomer and the molecular ion observed. HRF = heterocyclic ring fission and PC = procyanidin, Figure S5: An UV chromatogram at 280 nm, total ion chromatogram (TIC) and a mass spectrum of the observed proanthocyanidin (PA) hump of the PA fraction A-PC 2 obtained from the ultrahigh-resolution MS analysis. AU = absorbance unit, G = galloyl group, NL = normalized intensity, PC = procyanidin, PD = prodelphinidin, RT = retention time (min), Table S5: Characterization of the main ions of the proanthocyanidin (PA) fraction A-PC 2 obtained from the ultrahigh-resolution MS analysis: the degree of polymerization (DP), molecular formulae, calculated exact mass, the monomeric composition of the PA oligomer and the molecular ion observed. HRF = heterocyclic ring fission, PC = procyanidin, and QM = quinone-methide cleavage, Figure S6: An UV chromatogram at 280 nm, total ion chromatogram (TIC) and a mass spectrum of the observed proanthocyanidin (PA) hump of the PA fraction A-PC 3 obtained from the ultrahigh-resolution MS analysis. AU = absorbance unit, G = galloyl group, NL = normalized intensity, PC = procyanidin, PD = prodelphinidin, RT = retention time (min), Table S6: Characterization of the main ions of the proanthocyanidin (PA) fraction A-PC 3 obtained from the ultrahigh-resolution MS analysis: the degree of polymerization (DP), molecular formulae, calculated exact mass, the monomeric composition of the PA oligomer and the molecular ion observed. HRF = heterocyclic ring fission, PC = procyanidin, and QM = quinone-methide cleavage, Figure S7: An UV chromatogram at 280 nm, total ion chromatogram (TIC) and a mass spectrum of the observed proanthocyanidin (PA) hump of the PA fraction B-PD 1 obtained from the ultrahigh-resolution MS analysis. AU = absorbance unit, G = galloyl group, NL = normalized intensity, PC = procyanidin, PD = prodelphinidin, RT = retention time (min), Table S7: Characterization of the main ions of the proanthocyanidin (PA) fraction B-PD 1 obtained from the ultrahigh-resolution MS analysis: the degree of polymerization (DP), molecular formulae, calculated exact mass, the monomeric composition of the PA oligomer and the molecular ion observed. PD = prodelphinidin, Figure S8: An UV chromatogram at 280 nm, total ion chromatogram (TIC) and a mass spectrum of the observed proanthocyanidin (PA) hump of the PA fraction B-PD 2 obtained from the ultrahigh-resolution MS analysis. AU = absorbance unit, G = galloyl group, NL = normalized intensity, PC = procyanidin, PD = prodelphinidin, RT = retention time (min), Table S8: Characterization of the main ions of the proanthocyanidin (PA) fraction B-PD 2 obtained from the ultrahigh-resolution MS analysis: the degree of polymerization (DP), molecular formulae, calculated exact mass, the monomeric composition of the PA oligomer and the molecular ion observed. PD = prodelphinidin, Figure S9: An UV chromatogram at 280 nm, total ion chromatogram (TIC) and a mass spectrum of the observed proanthocyanidin (PA) hump of the PA fraction B-PD 3 obtained from the ultrahigh-resolution MS analysis. AU = absorbance unit, G = galloyl group, NL = normalized intensity, PC = procyanidin, PD = prodelphinidin, RT = retention time (min), Table S9: Characterization of the main ions of the proanthocyanidin (PA) fraction B-PD 3 obtained from the ultrahigh-resolution MS analysis: the degree of polymerization (DP), molecular formulae, calculated exact mass, the monomeric composition of the PA oligomer and the molecular ion observed. PD = prodelphinidin and QM = quinone-methide cleavage, Figure S10: An UV chromatogram at 280 nm, total ion chromatogram (TIC) and a mass spectrum of the observed proanthocyanidin (PA) hump of the PA fraction B-PD 4 obtained from the ultrahigh-resolution MS analysis. AU = absorbance unit, G = galloyl group, NL = normalized intensity, PC = procyanidin, PD = prodelphinidin, RT = retention time (min), Table S10: Characterization of the main ions of the proanthocyanidin (PA) fraction B-PD 4 obtained from the ultrahigh-resolution MS analysis: the degree of polymerization (DP), molecular formulae, calculated exact mass, the monomeric composition of the PA oligomer and the molecular ion observed. PD = prodelphinidin and QM = quinone-methide cleavage, Figure S11: An UV chromatogram at 280 nm, total ion chromatogram (TIC) and a mass spectrum of the observed proanthocyanidin (PA) hump of the PA fraction B-PC 1 obtained from the ultrahigh-resolution MS analysis. AU = absorbance unit, G = galloyl group, NL = normalized intensity, PC = procyanidin, PD = prodelphinidin, RT = retention time (min), Table S11: Characterization of the main ions of the proanthocyanidin (PA) fraction B-PC 1 obtained from the ultrahigh-resolution MS analysis: the degree of polymerization (DP), molecular formulae, calculated exact mass, the monomeric composition of the PA oligomer and the molecular ion observed. PC = procyanidin and PD = prodelphinidin, Figure S12: An UV chromatogram at 280 nm, total ion chromatogram (TIC) and a mass spectrum of the observed proanthocyanidin (PA) hump of the PA fraction B-PC 2 obtained from the ultrahigh-resolution MS analysis. AU = absorbance unit, G = galloyl group, NL = normalized intensity, PC = procyanidin, PD = prodelphinidin, RT = retention time (min), Table S12: Characterization of the main ions of the proanthocyanidin (PA) fraction B-PC 2 obtained from the ultrahigh-resolution MS analysis: the degree of polymerization (DP), molecular formulae, calculated exact mass, the monomeric composition of the PA oligomer and the molecular ion observed. PC = procyanidin, PD = prodelphinidin, QM = quinone-methide cleavage, Figure S13: An UV chromatogram at 280 nm, total ion chromatogram (TIC) and a mass spectrum of the observed proanthocyanidin (PA) hump of the PA fraction B-PC-G 2 obtained from the ultrahigh-resolution MS analysis. AU = absorbance unit, G = galloyl group, NL = normalized intensity, PC = procyanidin, PD = prodelphinidin, RT = retention time (min), Table S13: Characterization of the main ions of the proanthocyanidin (PA) fraction B-PC-G 2 obtained from the ultrahigh-resolution MS analysis: the degree of polymerization (DP), molecular formulae, calculated exact mass, the monomeric composition of the PA oligomer and the molecular ion observed. FL = flavonoid, G = galloyl group, PC = procyanidin, PD = prodelphinidin, QM = quinone-methide cleavage, RDA = retro-Diels–Alder, Figure S14: An UV chromatogram at 280 nm, total ion chromatogram (TIC) and a mass spectrum of the observed proanthocyanidin (PA) hump of the PA fraction B-PC/PD obtained from the ultrahigh-resolution MS analysis. AU = absorbance unit, G = galloyl group, NL = normalized intensity, PC = procyanidin, PD = prodelphinidin, RT = retention time (min), Table S14: Characterization of the main ions of the proanthocyanidin (PA) fraction B-PC/PD obtained from the ultrahigh-resolution MS analysis: the degree of polymerization (DP), molecular formulae, calculated exact mass, the monomeric composition of the PA oligomer and the molecular ion observed. PC = procyanidin, PD = prodelphinidin, QM = quinone-methide cleavage, Figure S15: Comparison of observed enthalpies (Δ*H*_obs_) from the proanthocyanidin fractions with the effect of the degree of polymerization taken into account (A) and a close-up on the smaller enthalpy changes (B). Fractions from the same plant source are marked in the same symbols and fractions containing A-type linkages are marked with symbols with an open center. For an explanation of the fraction codes, please refer to the original article.

## References

[1] Esatbeyoglu T., Winterhalter P. (2010). Preparation of Dimeric Procyanidins B1, B2, B5, and B7 from a Polymeric Procyanidin Fraction of Black Chokeberry (*Aronia Melanocarpa*). J. Agric. Food Chem..

[2] Esatbeyoglu T., Wray V., Winterhalter P. (2015). Isolation of Dimeric, Trimeric, Tetrameric and Pentameric Procyanidins from Unroasted Cocoa Beans (*Theobroma Cacao* L.) Using Countercurrent Chromatography. Food Chem..

[3] Ito C., Oki T., Yoshida T., Nanba F., Yamada K., Toda T. (2013). Characterisation of Proanthocyanidins from Black Soybeans: Isolation and Characterisation of Proanthocyanidin Oligomers from Black Soybean Seed Coats. Food Chem..

[4] Köhler N., Wray V., Winterhalter P. (2008). Preparative Isolation of Procyanidins from Grape Seed Extracts by High-Speed Counter-Current Chromatography. J. Chromatogr. A.

[5] Leppä M.M., Karonen M., Tähtinen P., Engström M.T., Salminen J.P. (2018). Isolation of Chemically Well-Defined Semipreparative Liquid Chromatography Fractions from Complex Mixtures of Proanthocyanidin Oligomers and Polymers. J. Chromatogr. A.

[6] Hagerman A.E. (2012). Fifty Years of Polyphenol-Protein Complexes. In Recent Advances in Polyphenol Research.

[7] Hansen T.V.A., Fryganas C., Acevedo N., Caraballo L., Thamsborg S.M., Mueller-Harvey I., Williams A.R. (2016). Proanthocyanidins Inhibit *Ascaris Suum* Glutathione-S-Transferase Activity and Increase Susceptibility of Larvae to Levamisole in Vitro. Parasitol. Int..

[8] Butter N.L., Dawson J.M., Wakelin D., Buttery P.J. (2001). Effect of Dietary Condensed Tannins on Gastrointestinal Nematodes. J. Agric. Sci..

[9] Klongsiriwet C., Quijada J., Williams A.R., Mueller-Harvey I., Williamson E.M., Hoste H. (2015). Synergistic Inhibition of *Haemonchus Contortus* Exsheathment by Flavonoid Monomers and Condensed Tannins. Int. J. Parasitol. Drugs Drug Resist..

[10] Desrues O., Fryganas C., Ropiak H.M., Mueller-Harvey I., Enemark H.L., Thamsborg S.M. (2016). Impact of Chemical Structure of Flavanol Monomers and Condensed Tannins on in Vitro Anthelmintic Activity against Bovine Nematodes. Parasitology.

[11] Novobilský A., Stringano E., Hayot Carbonero C., Smith L.M.J.J., Enemark H.L., Mueller-Harvey I., Thamsborg S.M. (2013). In Vitro Effects of Extracts and Purified Tannins of Sainfoin (*Onobrychis viciifolia*) against Two Cattle Nematodes. Vet. Parasitol..

[12] Quijada J., Fryganas C., Ropiak H.M., Ramsay A., Mueller-Harvey I., Hoste H. (2015). Anthelmintic Activities against *Haemonchus Contortus* or *Trichostrongylus Colubriformis* from Small Ruminants Are Influenced by Structural Features of Condensed Tannins. J. Agric. Food Chem..

[13] Williams A.R., Ropiak H.M., Fryganas C., Desrues O., Mueller-Harvey I., Thamsborg S.M. (2014). Assessment of the Anthelmintic Activity of Medicinal Plant Extracts and Purified Condensed Tannins against Free-Living and Parasitic Stages of *Oesophagostomum Dentatum*. Parasit. Vectors.

[14] Azuhnwi B.N., Hertzberg H., Arrigo Y., Gutzwiller A., Hess H.D., Mueller-Harvey I., Torgerson P.R., Kreuzer M., Dohme-Meier F. (2013). Investigation of Sainfoin (*Onobrychis viciifolia*) Cultivar Differences on Nitrogen Balance and Fecal Egg Count in Artificially Infected Lambs. J. Anim. Sci..

[15] Mueller-Harvey I., Bee G., Dohme-Meier F., Hoste H., Karonen M., Kölliker R., Lüscher A., Niderkorn V., Pellikaan W.F., Salminen J.P. (2019). Benefits of Condensed Tannins in Forage Legumes Fed to Ruminants: Importance of Structure, Concentration, and Diet Composition. Crop Sci..

[16] Min B.R., Hart S.P. (2003). Tannins for Suppression of Internal Parasites. J. Anim. Sci..

[17] Waghorn G.C., McNabb W.C. (2003). Consequences of Plant Phenolic Compounds for Productivity and Health of Ruminants. Proc. Nutr. Soc..

[18] Min B.R., Solaiman S., Gurung N., Behrends J., Eun J.S., Taha E., Rose J. (2012). Effects of Pine Bark Supplementation on Performance, Rumen Fermentation, and Carcass Characteristics of Kiko Crossbred Male Goats. J. Anim. Sci..

[19] Hoste H., Torres-Acosta J.F., Alonso-diaz M.Á., Brunet S., Sandoval-Castro C., Adote S.H. (2008). Identification and Validation of Bioactive Plants for the Control of Gastrointestinal Nematodes in Small Ruminants. Trop. Biomed..

[20] Hoste H., Martinez-Ortiz-De-Montellano C., Manolaraki F., Brunet S., Ojeda-Robertos N., Fourquaux I., Torres-Acosta J.F.J., Sandoval-Castro C.A. (2012). Direct and Indirect Effects of Bioactive Tannin-Rich Tropical and Temperate Legumes against Nematode Infections. Vet. Parasitol..

[21] Malisch C.S., Lüscher A., Baert N., Engström M.T., Studer B., Fryganas C., Suter D., Mueller-Harvey I., Salminen J.P. (2015). Large Variability of Proanthocyanidin Content and Composition in Sainfoin (*Onobrychis viciifolia*). J. Agric. Food Chem..

[22] Sottie E.T., Acharya S.N., McAllister T., Thomas J., Wang Y., Iwaasa A. (2014). Alfalfa Pasture Bloat Can Be Eliminated by Intermixing with Newly-Developed Sainfoin Population. Agron. J..

[23] Shalaby H.A. (2013). Anthelmintics Resistance; How to Overcome It?. Iran. J. Parasitol..

[24] Rose Vineer H., Morgan E.R., Hertzberg H., Bartley D.J., Bosco A., Charlier J., Chartier C., Claerebout E., De Waal T., Hendrickx G. (2020). Increasing Importance of Anthelmintic Resistance in European Livestock: Creation and Meta-Analysis of an Open Database. Parasite.

[25] Ahuir-Baraja A.E., Cibot F., Llobat L., Garijo M.M. (2021). Anthelmintic Resistance: Is a Solution Possible?. Exp. Parasitol..

[26] Lanusse C., Lifschitz A., Alvarez L. (2015). Basic and Clinical Pharmacology Contribution to Extend Anthelmintic Molecules Lifespan. Vet. Parasitol..

[27] Alvarez L., Lifschitz A., Entrocasso C., Manazza J., Mottier L., Borda B., Virkel G., Lanusse C. (2008). Evaluation of the Interaction between Ivermectin and Albendazole Following Their Combined Use in Lambs. J. Vet. Pharmacol. Ther..

[28] Armstrong S.A., Klein D.R., Whitney T.R., Scott C.B., Muir J.P., Lambert B.D., Craig T.M. (2013). Effect of Using Redberry Juniper (*Juniperus Pinchotii*) to Reduce *Haemonchus Contortus* in Vitro Motility and Increase Ivermectin Efficacy. Vet. Parasitol..

[29] Whitney T.R., Wildeus S., Zajac A.M. (2013). The Use of Redberry Juniper (*Juniperus Pinchotii*) to Reduce *Haemonchus Contortus* Fecal Egg Counts and Increase Ivermectin Efficacy. Vet. Parasitol..

[30] Gaudin E., Simon M., Quijada J., Schelcher F., Sutra J.F., Lespine A., Hoste H. (2016). Efficacy of Sainfoin (*Onobrychis viciifolia*) Pellets against Multi Resistant *Haemonchus Contortus* and Interaction with Oral Ivermectin: Implications for on-Farm Control. Vet. Parasitol..

[31] Malsa J., Courtot É., Boisseau M., Dumont B., Gombault P., Kuzmina T.A., Basiaga M., Lluch J., Annonay G., Dhorne-Pollet S. (2022). Effect of Sainfoin (*Onobrychis viciifolia*) on Cyathostomin Eggs Excretion, Larval Development, Larval Community Structure and Efficacy of Ivermectin Treatment in Horses. Parasitology.

[32] Sillanpää M., Engström M.T., Tähtinen P., Green R.J., Käpylä J., Näreaho A., Karonen M. (2023). Tannins Can Have Direct Interactions with Anthelmintics: Investigations by Isothermal Titration Calorimetry. Molecules.

[33] Karonen M., Bin Imran I., Engström M.T., Salminen J.P. (2021). Characterization of Natural and Alkaline-Oxidized Proanthocyanidins in Plant Extracts by Ultrahigh-Resolution UHPLC-MS/MS. Molecules.

[34] Engström M.T., Pälijärvi M., Fryganas C., Grabber J.H., Mueller-Harvey I., Salminen J.P. (2014). Rapid Qualitative and Quantitative Analyses of Proanthocyanidin Oligomers and Polymers by UPLC-MS/MS. J. Agric. Food Chem..

[35] Suominen E., Savila S., Sillanpää M., Damlin P., Karonen M. (2023). Affinity of Tannins to Cellulose: A Chromatographic Tool for Revealing Structure-Activity Patterns. Molecules.

[36] Sui Y., Zheng Y., Li X., Li S., Xie B., Sun Z. (2016). Characterization and Preparation of Oligomeric Procyanidins from *Litchi Chinensis* Pericarp. Fitoterapia.

[37] Karonen M., Loponen J., Ossipov V., Pihlaja K. (2004). Analysis of Procyanidins in Pine Bark with Reversed-Phase and Normal-Phase High-Performance Liquid Chromatography-Electrospray Ionization Mass Spectrometry. Anal. Chim. Acta.

[38] Lin L.Z., Sun J., Chen P., Monagas M.J., Harnly J.M. (2014). UHPLC-PDA-ESI/HRMS n profiling Method to Identify and Quantify Oligomeric Proanthocyanidins in Plant Products. J. Agric. Food Chem..

[39] Li S., Xiao J., Chen L., Hu C., Chen P., Xie B., Sun Z. (2012). Identification of A-Series Oligomeric Procyanidins from Pericarp of *Litchi Chinensis* by FT-ICR-MS and LC-MS. Food Chem..

[40] Turnbull W.B., Daranas A.H. (2003). On the Value of c: Can Low Affinity Systems Be Studied by Isothermal Titration Calorimetry?. J. Am. Chem. Soc..

[41] Tellinghuisen J. (2004). Statistical Error in Isothermal Titration Calorimetry. Methods Enzymol..

[42] Tellinghuisen J. (2008). Stupid Statistics!. Methods Cell Biol..

[43] Frazier R.A., Deaville E.R., Green R.J., Stringano E., Willoughby I., Plant J., Mueller-Harvey I. (2010). Interactions of Tea Tannins and Condensed Tannins with Proteins. J. Pharm. Biomed. Anal..

[44] Tsuchiya H. (1999). Effects of Green Tea Catechins on Membrane Fluidity. Pharmacology.

[45] Zeller W.E., Reinhardt L.A., Robe J.T., Sullivan M.L., Panke-Buisse K. (2020). Comparison of Protein Precipitation Ability of Structurally Diverse Procyanidin-Rich Condensed Tannins in Two Buffer Systems. J. Agric. Food Chem..

[46] Leppä M.M., Laitila J.E., Salminen J.P. (2020). Distribution of Protein Precipitation Capacity within Variable Proanthocyanidin Fingerprints. Molecules.

[47] Kilmister R.L., Faulkner P., Downey M.O., Darby S.J., Falconer R.J. (2016). The Complexity of Condensed Tannin Binding to Bovine Serum Albumin—An Isothermal Titration Calorimetry Study. Food Chem..

[48] Harbertson J.F., Kilmister R.L., Kelm M.A., Downey M.O. (2014). Impact of Condensed Tannin Size as Individual and Mixed Polymers on Bovine Serum Albumin Precipitation. Food Chem..

[49] Cala O., Pinaud N., Simon C., Fouquet E., Laguerre M., Dufourc E.J., Pianet I. (2010). NMR and Molecular Modeling of Wine Tannins Binding to Saliva Proteins: Revisiting Astringency from Molecular and Colloidal Prospects. Faseb J..

[50] Hagerman A.E., Rice M.E., Ritchard N.T. (1998). Mechanisms of Protein Precipitation for Two Tannins, Pentagalloyl Glucose and Epicatechin16 (4 → 8) Catechin (Procyanidin). J. Agric. Food Chem..

[51] Simon C., Barathieu K., Laguerre M., Schmitter J.M., Fouquet E., Pianet I., Dufourc E.J. (2003). Three-Dimensional Structure and Dynamics of Wine Tannin-Saliva Protein Complexes. A Multitechnique Approach. Biochemistry.

[52] Tang H.R., Covington A.D., Hancock R.A. (2003). Structure–Activity Relationships in the Hydrophobic Interactions of Polyphenols with Cellulose and Collagen. Biopolymers.

[53] Virtanen V., Green R.J., Karonen M. (2022). Interactions between Hydrolysable Tannins and Lipid Vesicles from *Escherichia Coli* with Isothermal Titration Calorimetry. Molecules.

[54] Poncet-Legrand C., Gautier C., Cheynier V., Imberty A. (2007). Interactions between Flavan-3-Ols and Poly(L-Proline) Studied by Isothermal Titration Calorimetry: Effect of the Tannin Structure. J. Agric. Food Chem..

[55] Soares S., García-Estévez I., Ferrer-Galego R., Brás N.F., Brandão E., Silva M., Teixeira N., Fonseca F., Sousa S.F., Ferreira-da-Silva F. (2018). Study of Human Salivary Proline-Rich Proteins Interaction with Food Tannins. Food Chem..

[56] McRae J.M., Falconer R.J., Kennedy J.A. (2010). Thermodynamics of Grape and Wine Tannin Interaction with Polyproline: Implications for Red Wine Astringency. J. Agric. Food Chem..

[57] Ropiak H.M., Lachmann P., Ramsay A., Green R.J., Mueller-Harvey I. (2017). Identification of Structural Features of Condensed Tannins That Affect Protein Aggregation. PLoS ONE.

[58] Frazier R.A., Papadopoulou A., Mueller-Harvey I., Kissoon D., Green R.J. (2003). Probing Protein-Tannin Interactions by Isothermal Titration Microcalorimetry. J. Agric. Food Chem..

[59] Karonen M., Oraviita M., Mueller-Harvey I., Salminen J.-P., Green R.J. (2015). Binding of an Oligomeric Ellagitannin Series to Bovine Serum Albumin (BSA): Analysis by Isothermal Titration Calorimetry (ITC). J. Agric. Food Chem.

[60] Sun Q., He J., Yang H., Li S., Zhao L., Li H. (2017). Analysis of Binding Properties and Interaction of Thiabendazole and Its Metabolite with Human Serum Albumin via Multiple Spectroscopic Methods. Food Chem..

[61] Stepniak A., Erdenebayar B., Biernacka M., Buczkowski A., Zavodnik L., Zavodnik I., Palecz B. (2020). Calorimetric Studies of α-Cyclodextrin Inclusion Complexes with Carbendazim and Thiabendazole. Phys. Chem. Liq..

[62] Zhu W., Wang R.F., Khalifa I., Li C.M. (2019). Understanding toward the Biophysical Interaction of Polymeric Proanthocyanidins (Persimmon Condensed Tannins) with Biomembranes: Relevance for Biological Effects. J. Agric. Food Chem..

[63] Engström M.T., Pälijärvi M., Salminen J.P. (2015). Rapid Fingerprint Analysis of Plant Extracts for Ellagitannins, Gallic Acid, and Quinic Acid Derivatives and Quercetin-, Kaempferol- and Myricetin-Based Flavonol Glycosides by UPLC-QqQ-MS/MS. J. Agric. Food Chem..

[64] Gea A., Stringano E., Brown R.H., Mueller-Harvey I. (2011). In Situ Analysis and Structural Elucidation of Sainfoin (*Onobrychis viciifolia*) Tannins for High-Throughput Germplasm Screening. J. Agric. Food Chem..

