# Supplementary material for: Exploring the Interactions between Plant Proanthocyanidins and Thiabendazole: Insights from Isothermal Titration Calorimetry
Source: Molecules. 2024 Jul 25;29(15):3492. doi: 10.3390/molecules29153492 (PMC11313799; doi:10.3390/molecules29153492)
Supplement: Supplementary file 1 [file molecules-29-03492-s001.zip › molecules-3025346-supplementary.pdf]

molecules

Supplementary material

Exploring the Interactions between Plant Proanthocyanidins and Thiabendazole: Insights from Isothermal Titration Calorimetry

Mimosa Sillanpää [1,*], Marica T. Engström [2], Petri Tähtinen [1], Rebecca J. Green [3], Jarmo Käpylä [4], Anu Näreaho [5] and Maarit Karonen [1]

[1] Department of Chemistry, University of Turku, FI-20014 Turku, Finland; peppe@utu.fi (P.T.); maarit.karonen@utu.fi (M.K.)
[2] Institute of Biomedicine, University of Turku, FI-20014 Turku, Finland; mtengs@utu.fi
[3] School of Chemistry, Food and Pharmacy, University of Reading, Whiteknights, P.O. Box 224, Reading RG6 6AP, UK; rebecca.green@reading.ac.uk
[4] Department of Life Technologies, University of Turku, FI-20014 Turku, Finland; jakapy@utu.fi
[5] Department of Veterinary Biosciences, University of Helsinki, FI-00014 Helsinki, Finland; anu.nareaho@helsinki.fi
* Correspondence: mamsil@utu.fi

Table of Contents

Figure S1 2
Table S1 2
Figure S2 3
Table S2 3
Figure S3 5
Table S3 5
Figure S4 6
Table S4 6
Figure S5 7
Table S5 7
Figure S6 8
Table S6 9
Figure S7 10
Table S7 10
Figure S8 11
Table S8 11
Figure S9 12
Table S9 12

Figure S10 13

Table S10 13

Figure S11 14

Table S11 14

Figure S12 15

Table S12 15

Figure S13 17

Table S13 18

Figure S14 19

Table S14 19

Figure S15 20

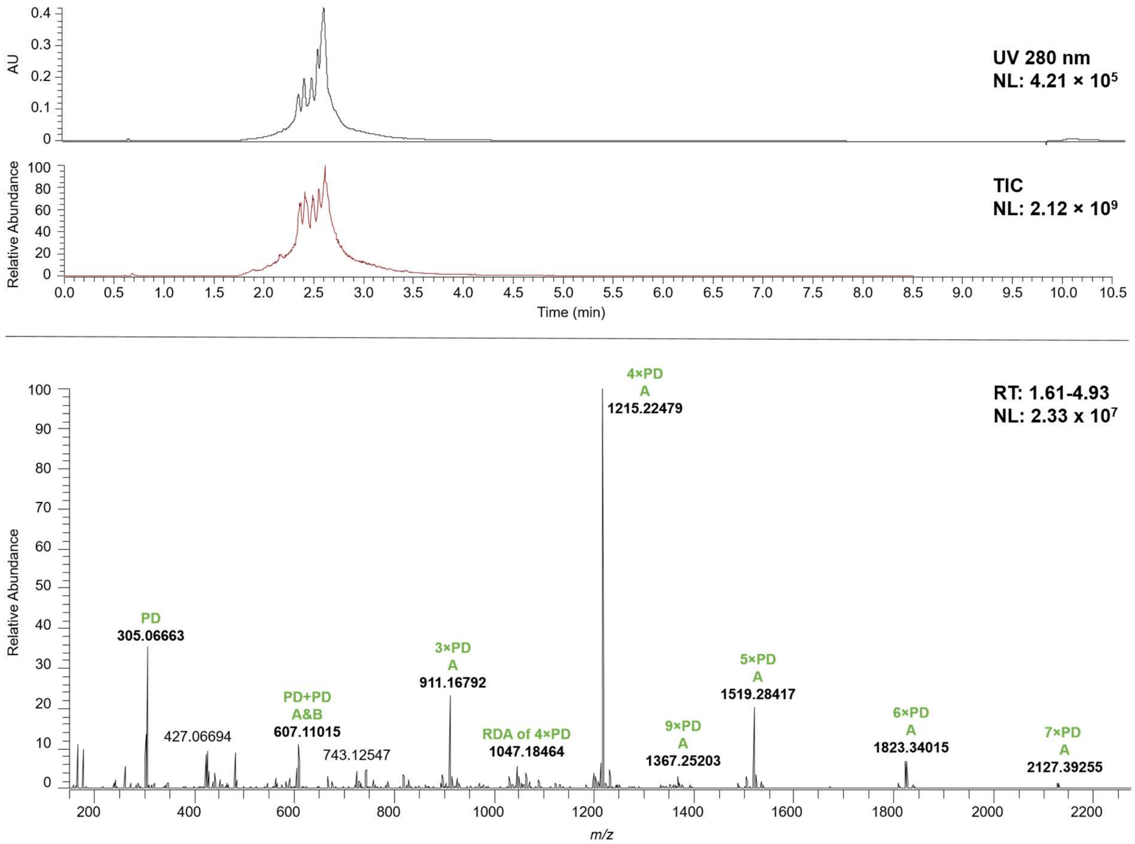

Figure S1. An UV chromatogram at 280 nm, total ion chromatogram (TIC) and a mass spectrum of the observed proanthocyanidin (PA) hump of the PA fraction A-PD 1 obtained from the ultra-high-resolution MS analysis. AU=absorbance unit, G=galloyl group, NL=normalised intensity, PC=procyanidin, PD=prodelphinidin, RT=retention time (min).

Table S1. Characterization of the main ions of the proanthocyanidin (PA) fraction A-PD 1 obtained from the ultrahigh-resolution MS analysis: the degree of polymerization (DP), molecular formulae, calculated exact mass, the monomeric composition of the PA oligomer and the molecular ion observed. PC=procyanidin, PD=prodelphinidin, and RDA= retro-Diels-Alder.

DP	Molecular formula	Mcalculated	Monomeric composition	No. of A-type bonds	$[M-H]^-$	$[M-2H]^{2-}$
1	$C_{15}H_{14}O_7$	306.07396	PD	0	305.06663	-
2	$C_{30}H_{24}O_{14}$	608.11661	2×PD	1	607.11015	-
2	$C_{30}H_{26}O_{14}$	610.13226	2×PD	0	609.12496	-
3	$C_{45}H_{36}O_{21}$	912.17492	3×PD	1	911.16792	-
4	$C_{52}H_{40}O_{24}$	1048.19096	RDA fragment of 4×PD	0	1047.18464	-
4	$C_{60}H_{48}O_{28}$	1216.23322	4×PD	1	1215.22479	-
5	$C_{75}H_{60}O_{35}$	1520.29153	5×PD	1	1519.28417	-
6	$C_{90}H_{72}O_{42}$	1824.34983	6×PD	1	1823.34015	-
7	$C_{105}H_{84}O_{49}$	2128.40814	7×PD	1	2127.39255	-
9	$C_{135}H_{108}O_{63}$	2736.52475	9×PD	1	-	1367.25203

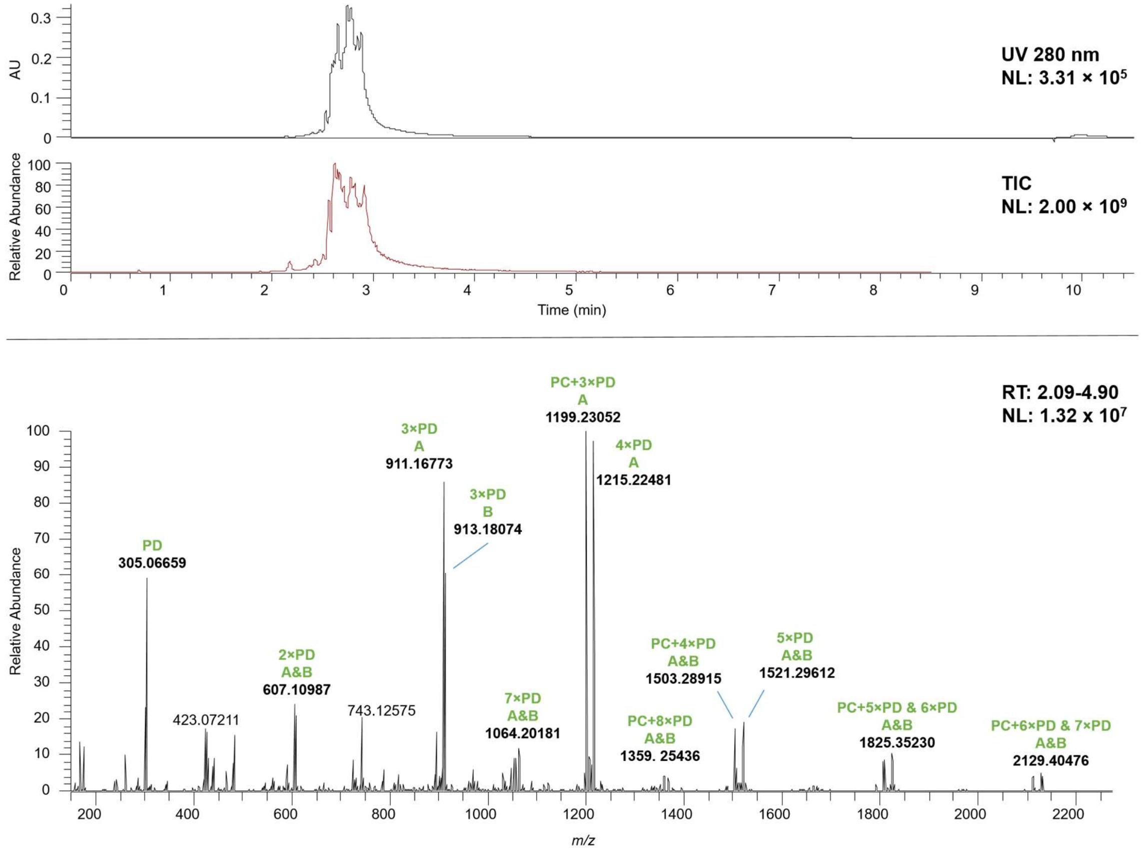

Figure S2. An UV chromatogram at 280 nm, total ion chromatogram (TIC) and a mass spectrum of the observed proanthocyanidin (PA) hump of the PA fraction A-PD 2 obtained from the ultra-high-resolution MS analysis. AU=absorbance unit, G=galloyl group, NL=normalised intensity, PC=procyanidin, PD=prodelphinidin, RT=retention time (min).

Table S2. Characterization of the main ions of the proanthocyanidin (PA) fraction A-PD 2 obtained from the ultrahigh-resolution MS analysis: the degree of polymerization (DP), molecular formulae, calculated exact mass, the monomeric composition of the PA oligomer and the molecular ion observed. PC=procyanidin and PD=prodelphinidin.

DP	Molecular formula	Mcalculated	Monomeric composition	No. of A-type bonds	$[M-H]^-$	$[M-2H]^{2-}$
1	$C_{15}H_{14}O_7$	306.07396	PD	0	305.06659	-
2	$C_{30}H_{24}O_{14}$	608.11661	2×PD	1	607.10987	-
2	$C_{30}H_{26}O_{14}$	610.13226	2×PD	0	609.12481	-
3	$C_{45}H_{36}O_{21}$	912.17492	3×PD	1	911.16773	-
3	$C_{45}H_{38}O_{21}$	914.19057	3×PD	0	913.18074	-
4	$C_{60}H_{48}O_{27}$	1200.23831	PC+3×PD	1	1199.23052	-
4	$C_{60} H_{50} O_{27}$	1202.25396	PC+3×PD	0	1201.23952	-
4	$C_{60}H_{48}O_{28}$	1216.23322	4×PD	1	1215.22481	-
4	$C_{60}H_{50}O_{28}$	1218.24887	4×PD	0	1217.23367	-
5	$C_{75}H_{60}O_{34}$	1504.29661	PC+4×PD	1	1503.28915	-
5	$C_{75}H_{62}O_{35}$	1522.30718	5×PD	0	1521.29612	-
6	$C_{90}H_{72}O_{41}$	1808.35492	PC+5×PD	1	1807.34393	-
6	$C_{90}H_{74}O_{41}$	1810.37057	PC+5×PD	0	1807.34393	-
6	$C_{90}H_{72}O_{42}$	1824.34983	6×PD	1	1823.33851	-
6	$C_{90}H_{74}O_{42}$	1826.36548	6×PD	0	1825.35230	-
7	$C_{105}H_{84}O_{48}$	2112.41322	PC+6×PD	1	2111.40019	-
7	$C_{105}H_{86}O_{48}$	2114.42887	PC+6×PD	0	2113.40608	-
7	$C_{105}H_{84}O_{49}$	2128.40814	7×PD	1	2127.39061	-
7	$C_{105}H_{86}O_{49}$	2130.42379	7×PD	0	2129.40476	-
7	$C_{105}H_{84}O_{49}$	2128.40814	7×PD	1	-	1063.19610
7	$C_{105}H_{86}O_{49}$	2130.42379	7×PD	0	-	1064.20181
9	$C_{135}H_{108}O_{62}$	2720.52983	PC+8×PD	1	-	1359.25436
9	$C_{135}H_{110}O_{62}$	2720.52983	PC+8×PD	0	-	1360.25877

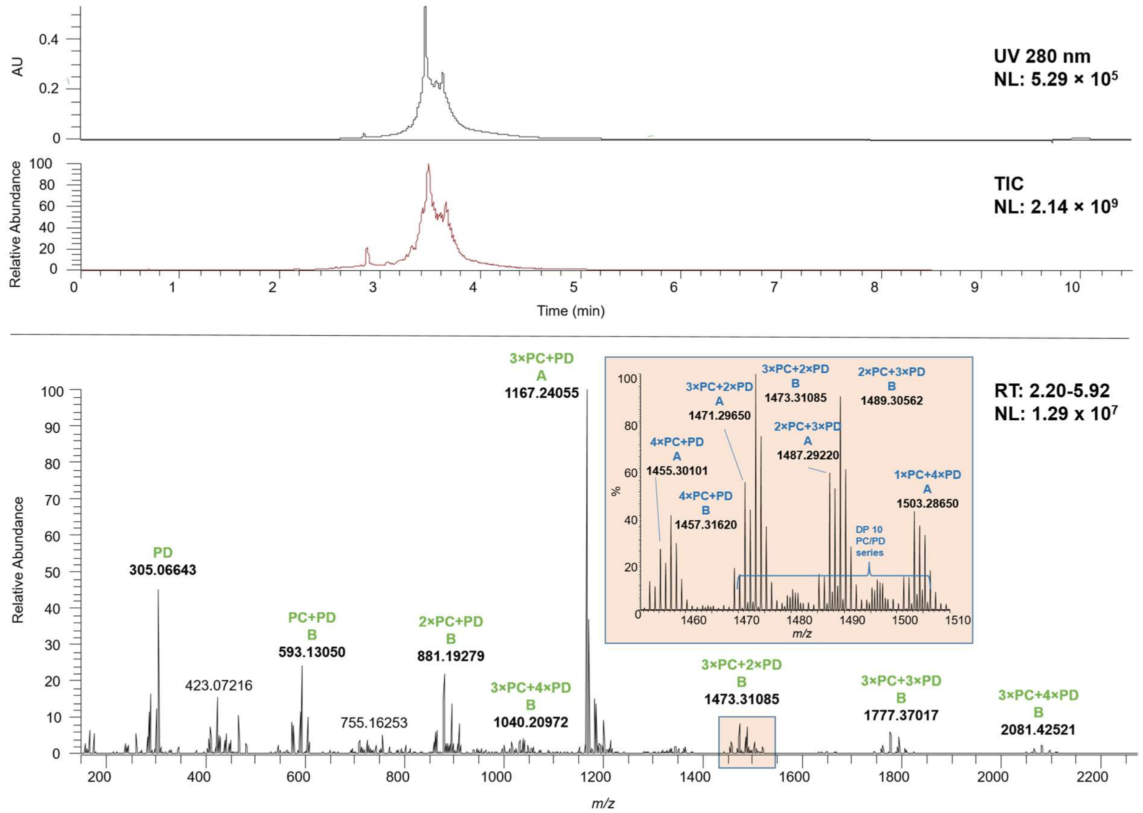

Figure S3. An UV chromatogram at 280 nm, total ion chromatogram (TIC) and a mass spectrum of the observed proanthocyanidin (PA) hump of the PA fraction A-PD 3 obtained from the ultra-high-resolution MS analysis. AU=absorbance unit, G=galloyl group, NL=normalised intensity, PC=procyanidin, PD=prodelphinidin, RT=retention time (min).

Table S3. Characterization of the main ions of the proanthocyanidin (PA) fraction A-PD 3 obtained from the ultrahigh-resolution MS analysis: the degree of polymerization (DP), molecular formulae, calculated exact mass, the monomeric composition of the PA oligomer and the molecular ion observed. PC=procyanidin and PD=prodelphinidin.

DP	Molecular formula	Mcalculated	Monomeric composition	No. of A-type bonds	$[M-H]^-$	$[M-2H]^{2-}$
1	$C_{15}H_{14}O_6$	290.07904	PC	0	289.07174	-
1	$C_{15}H_{14}O_7$	306.07396	PD	0	305.06643	-
2	$C_{30}H_{26}O_{13}$	594.13735	PC+PD	0	593.13050	-
3	$C_{45}H_{36}O_{19}$	880.18509	2×PC+PD	1	879.17740	-
3	$C_{45}H_{38}O_{19}$	882.20074	2×PC+PD	0	881.19279	-
3	$C_{45}H_{36}O_{20}$	896.18000	PC+2×PD	1	895.17315	-
3	$C_{45}H_{38}O_{20}$	898.19565	PC+2×PD	0	897.18592	-
4	$C_{60}H_{48}O_{25}$	1168.24848	3×PC+PD	1	1167.24055	-
4	$C_{60}H_{50}O_{25}$	1170.26413	3*PC+PD	0	1169.25525	-
4	$C_{60}H_{48}O_{26}$	1184.24339	2×PD+2×PD	1	1183.23596	-
5	$C_{75}H_{60}O_{32}$	1472.30678	3*PC+2*PD	1	1471.29650	-
5	$C_{75}H_{62}O_{32}$	1474.32243	3×PC+2×PD	0	1473.31085	-
5	$C_{75}H_{60}O_{33}$	1488.30170	2*PC+3*PD	1	1487.29220	-
5	$C_{75}H_{62}O_{33}$	1490.31735	2×PC+3×PD	0	1489.30562	-
6	$C_{90}H_{72}O_{39}$	1776.36509	3*PC+3*PD	1	1775.35383	-

6	$C_{90}H_{74}O_{39}$	1778.38074	3×PC+3×PD	0	1777.37017	-
6	$C_{90}H_{72}O_{40}$	1792.36000	3*PC+3*PD	1	1791.34992	-
6	$C_{90}H_{74}O_{40}$	1794.37565	2×PC+4×PD	0	1793.36394	-
7	$C_{105}H_{86}O_{46}$	2082.43904	3×PC+4×PD	0	2081.42521	-
7	$C_{105}H_{86}O_{46}$	2082.43904	3×PC+4×PD	0	-	1040.20972

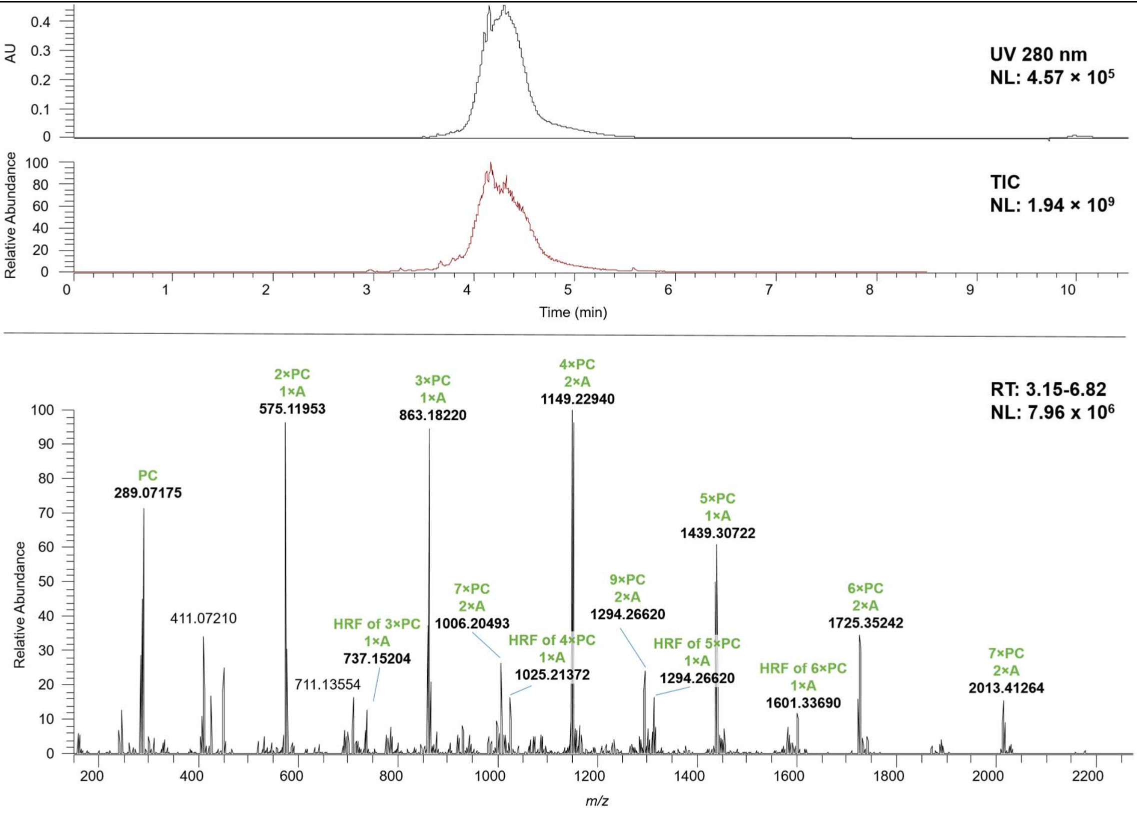

Figure S4. An UV chromatogram at 280 nm, total ion chromatogram (TIC) and a mass spectrum of the observed proanthocyanidin (PA) hump of the PA fraction A-PC 1 obtained from the ultra-high-resolution MS analysis. AU=absorbance unit, G=galloyl group, NL=normalised intensity, PC=procyanidin, PD=prodelphinidin, RT=retention time (min).

Table S4. Characterization of the main ions of the proanthocyanidin (PA) fraction A-PC 1 obtained from the ultrahigh-resolution MS analysis: the degree of polymerization (DP), molecular formulae, calculated exact mass, the monomeric composition of the PA oligomer and the molecular ion observed. HRF= heterocyclic ring fission and PC=procyanidin.

DP	Molecular formula	Mcalculated	Monomeric composition	No. of A-type bonds	$[M-H]^-$	$[M-2H]^{2-}$
1	$C_{15}H_{14}O_6$	290.07904	PC	0	289.07175	-
2	$C_{30}H_{24}O_{12}$	576.12678	2×PC	1	575.11953	-
3	$C_{39}H_{30}O_{15}$	738.15848	HRF fragment of 3×PC	1	737.15204	-
3	$C_{45}H_{34}O_{18}$	862.17452	3×PC	2	861.16762	-
3	$C_{45}H_{36}O_{18}$	864.19017	3×PC	1	863.18220	-
4	$C_{54}H_{42}O_{21}$	1026.22187	HRF fragment of 4×PC	1	1025.21372	-
4	$C_{60}H_{46}O_{24}$	1150.23791	4×PC	2	1149.22940	-
4	$C_{60}H_{48}O_{24}$	1152.25356	4×PC	1	1151.24321	-
5	$C_{69}H_{54}O_{27}$	1314.28526	HRF fragment of 5×PC	1	1313.27621	-
5	$C_{75}H_{58}O_{30}$	1438.30130	5×PC	2	1437.29284	-

5	$C_{75}H_{60}O_{30}$	1440.31695	5×PC	1	1439.30722	-
6	$C_{84}H_{66}O_{33}$	1602.34865	HRF fragment of 6×PC	1	1601.33690	-
6	$C_{90}H_{70}O_{36}$	1726.36469	6×PC	2	1725.35242	-
6	$C_{90}H_{72}O_{36}$	1728.38034	6×PC	1	1727.36447	-
6	$C_{90}H_{70}O_{36}$	1726.36469	6×PC	2	1725.35242	-
7	$C_{105}H_{82}O_{42}$	2014.42808	7×PC	2	-	1006.20493
7	$C_{105}H_{82}O_{42}$	2014.42808	7×PC	2	2013.41264	-
7	$C_{105}H_{84}O_{42}$	2016.44373	7×PC	1	2015.42358	-
9	$C_{135}H_{106}O_{54}$	2590.55486	9×PC	2	-	1294.26620
11	$C_{165}H_{130}O_{66}$	3166.68164	11×PC	2	-	1582.32820

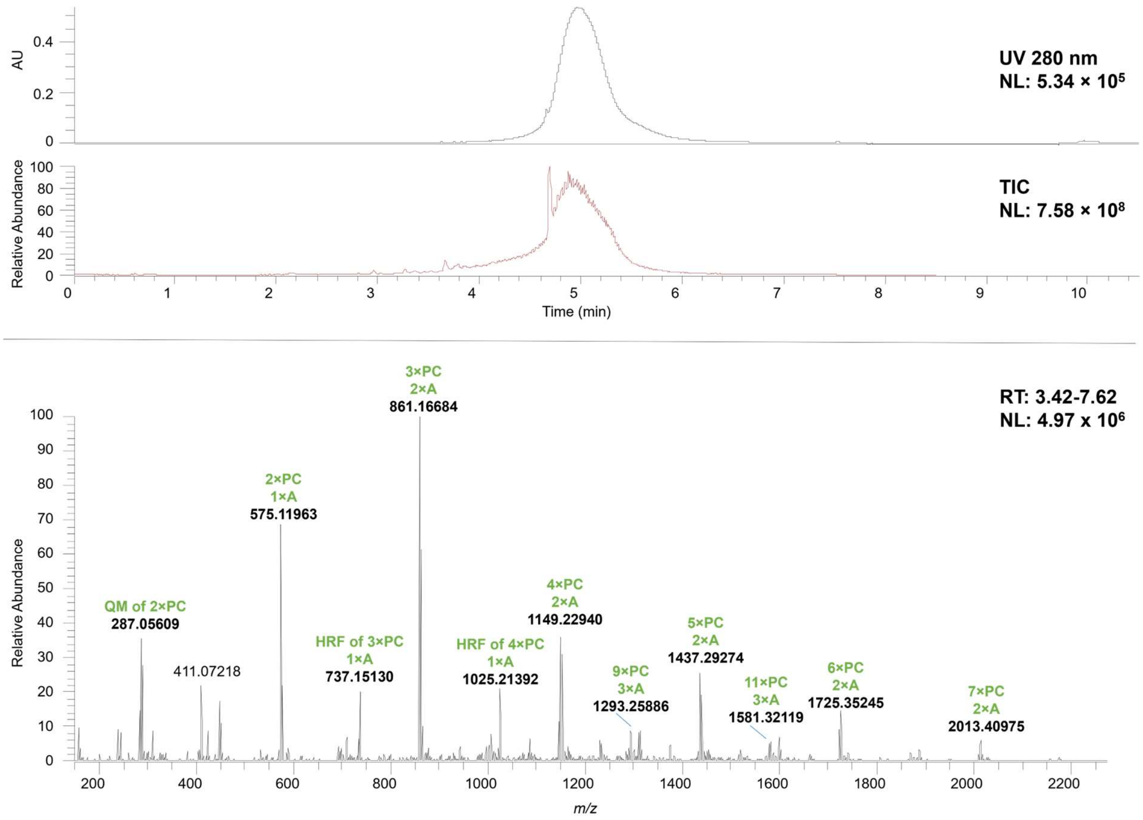

Figure S5. An UV chromatogram at 280 nm, total ion chromatogram (TIC) and a mass spectrum of the observed proanthocyanidin (PA) hump of the PA fraction A-PC 2 obtained from the ultra-high-resolution MS analysis. AU=absorbance unit, G=galloyl group, NL=normalised intensity, PC=procyanidin, PD=prodelphinidin, RT=retention time (min).

Table S5. Characterization of the main ions of the proanthocyanidin (PA) fraction A-PC 2 obtained from the ultrahigh-resolution MS analysis: the degree of polymerization (DP), molecular formulae, calculated exact mass, the monomeric composition of the PA oligomer and the molecular ion observed. HRF= heterocyclic ring fission, PC=procyanidin, and QM= quinone-methide cleavage.

DP	Molecular formula	Mcalculated	Monomeric composition	No. of A-type bonds	$[M-H]^-$	$[M-2H]^{2-}$
1	$C_{15}H_{12}O_6$	288.06339	QM fragment of 2×PC	0	287.05609	-
1	$C_{15}H_{14}O_6$	290.07904	PC	0	289.07194	-
2	$C_{30}H_{24}O_{12}$	576.12678	2×PC	1	575.11963	-

3	$C_{39}H_{30}O_{15}$	738.15848	HRF fragment of 3×PC	1	737.15130	-
3	$C_{45}H_{34}O_{18}$	862.17452	3×PC	2	861.16684	-
3	$C_{45}H_{36}O_{18}$	864.19017	3×PC	1	863.18065	-
4	$C_{54}H_{42}O_{21}$	1026.22187	HRF fragment of 4×PC	1	1025.21392	-
4	$C_{60}H_{46}O_{24}$	1150.23791	4×PC	2	1149.22940	-
4	$C_{60}H_{48}O_{24}$	1152.25356	4×PC	1	1151.24362	-
5	$C_{69}H_{54}O_{27}$	1314.28526	HRF fragment of 5×PC	1	1313.27416	-
5	$C_{69}H_{52}O_{27}$	1312.26961	HRF fragment of 5×PC	2	1311.25967	-
5	$C_{75}H_{56}O_{30}$	1436.28565	5×PC	3	1435.27878	-
5	$C_{75}H_{58}O_{30}$	1438.30130	5×PC	2	1437.29274	-
6	$C_{84}H_{64}O_{33}$	1600.33300	HRF fragment of 6×PC	2	1599.32191	-
6	$C_{84}H_{66}O_{33}$	1602.34865	HRF fragment of 6×PC	1	1601.33296	-
6	$C_{90}H_{68}O_{36}$	1724.34904	6×PC	3	1723.33901	-
6	$C_{90}H_{70}O_{36}$	1726.36469	6×PC	2	1725.35245	-
7	$C_{105}H_{80}O_{42}$	2012.41243	7×PC	3	2011.39830	-
7	$C_{105}H_{80}O_{42}$	2014.42808	7×PC	2	2013.40975	-
9	$C_{135}H_{104}O_{54}$	2588.53921	9×PC	3	-	1293.25886
11	$C_{165}H_{128}O_{66}$	3164.66599	11×PC	3	-	1581.32119

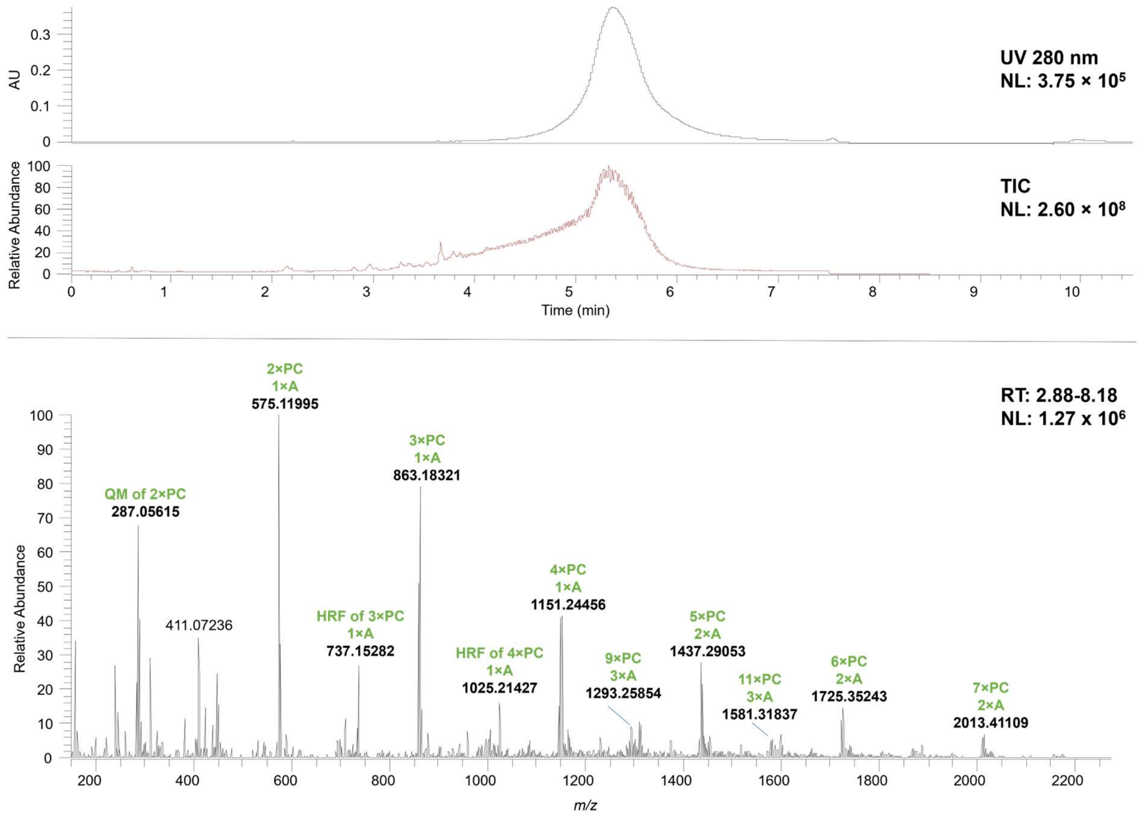

Figure S6. An UV chromatogram at 280 nm, total ion chromatogram (TIC) and a mass spectrum of the observed proanthocyanidin (PA) hump of the PA fraction A-PC 3 obtained from the ultra-high-resolution MS analysis. AU=absorbance unit, G=galloyl group, NL=normalised intensity, PC=procyanidin, PD=prodelphinidin, RT=retention time (min).

Table S6. Characterization of the main ions of the proanthocyanidin (PA) fraction A-PC 3 obtained from the ultrahigh-resolution MS analysis: the degree of polymerization (DP), molecular formulae, calculated exact mass, the monomeric composition of the PA oligomer and the molecular ion observed. HRF= heterocyclic ring fission, PC=procyanidin, and QM= quinone-methide cleavage.

DP	Molecular formula	Mcalculated	Monomeric composition	No. of A-type bonds	$[M-H]^-$	$[M-2H]^{2-}$
1	$C_{15}H_{12}O_6$	288.06339	QM fragment of 2×PC	0	287.05615	-
1	$C_{15}H_{14}O_6$	290.07904	PC	0	289.07182	-
2	$C_{30}H_{24}O_{12}$	576.12678	2×PC	1	575.11995	-
3	$C_{39}H_{30}O_{15}$	738.15848	HRF fragment of 3×PC	1	737.15282	-
3	$C_{45}H_{34}O_{18}$	862.17452	3×PC	2	861.16805	-
3	$C_{45}H_{36}O_{18}$	864.19017	3×PC	1	863.18321	-
4	$C_{54}H_{42}O_{21}$	1026.22187	HRF fragment of 4×PC	1	1025.21427	-
4	$C_{60}H_{46}O_{24}$	1150.23791	4×PC	2	1149.22970	-
4	$C_{60}H_{48}O_{24}$	1152.25356	4×PC	1	1151.24456	-
5	$C_{69}H_{52}O_{27}$	1312.26961	HRF fragment of 5×PC	2	1311.26115	-
5	$C_{69}H_{54}O_{27}$	1314.28526	HRF fragment of 5×PC	1	1313.27257	-
5	$C_{75}H_{58}O_{30}$	1438.30130	5×PC	2	1437.29053	-
5	$C_{75}H_{60}O_{30}$	1440.31695	5×PC	1	1439.30448	-
6	$C_{84}H_{64}O_{33}$	1600.33300	HRF fragment of 6×PC	2	1599.32241	-
6	$C_{84}H_{66}O_{33}$	1602.34865	HRF fragment of 6×PC	1	1601.33160	-
6	$C_{90}H_{70}O_{36}$	1726.36469	6×PC	2	1725.35243	-
7	$C_{105}H_{82}O_{42}$	2014.42808	7×PC	2	2013.41109	-
9	$C_{135}H_{104}O_{54}$	2588.53921	9×PC	3	-	1293.25854
11	$C_{165}H_{128}O_{66}$	3164.66599	11×PC	3	-	1581.31837

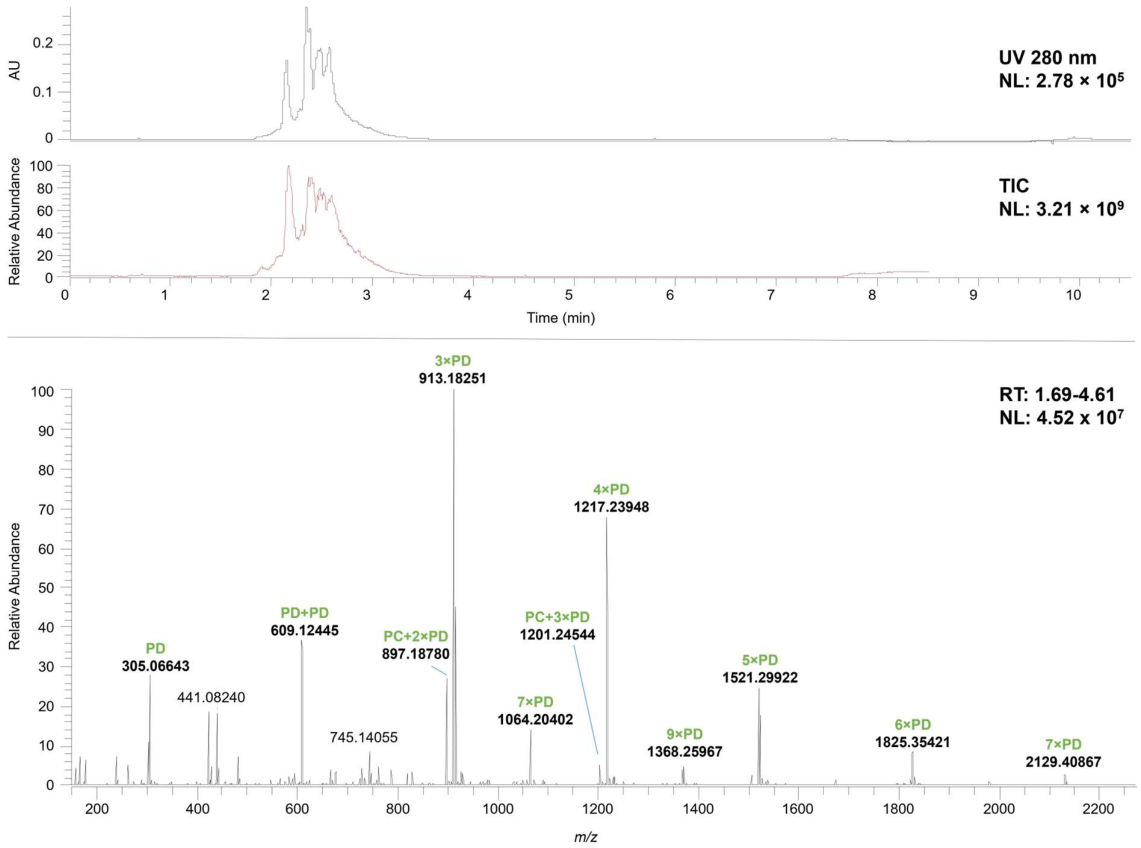

Figure S7. An UV chromatogram at 280 nm, total ion chromatogram (TIC) and a mass spectrum of the observed proanthocyanidin (PA) hump of the PA fraction B-PD 1 obtained from the ultra-high-resolution MS analysis. AU=absorbance unit, G=galloyl group, NL=normalised intensity, PC=procyanidin, PD=prodelphinidin, RT=retention time (min).

Table S7. Characterization of the main ions of the proanthocyanidin (PA) fraction B-PD 1 obtained from the ultrahigh-resolution MS analysis: the degree of polymerization (DP), molecular formulae, calculated exact mass, the monomeric composition of the PA oligomer and the molecular ion observed. PD=prodelphinidin.

DP	Molecular formula	Mcalculated	Monomeric composition	No. of A-type bonds	$[M\text{-}H]^-$	$[M\text{-}2H]^{2-}$
1	$C_{15}H_{14}O_7$	306.07396	PD	0	305.06643	-
2	$C_{30}H_{26}O_{14}$	610.13226	2×PD	0	609.12445	-
3	$C_{45}H_{38}O_{20}$	898.19565	PC+2×PD	0	897.18780	-
3	$C_{45}H_{38}O_{21}$	914.19057	3×PD	0	913.18251	-
4	$C_{60}H_{50}O_{27}$	1202.25396	PC+3×PD	0	1201.24544	-
4	$C_{60}H_{50}O_{28}$	1218.24887	4×PD	0	1217.23948	-
5	$C_{75}H_{62}O_{35}$	1522.30718	5×PD	0	1521.29922	-
6	$C_{90}H_{74}O_{42}$	1826.36548	6×PD	0	1825.35421	-
7	$C_{105}H_{86}O_{49}$	2130.42379	7×PD	0	-	1064.20402
7	$C_{105}H_{86}O_{49}$	2130.42379	7×PD	0	2129.40867	-
9	$C_{135}H_{110}O_{63}$	2738.54040	9×PD	0	-	1368.25967

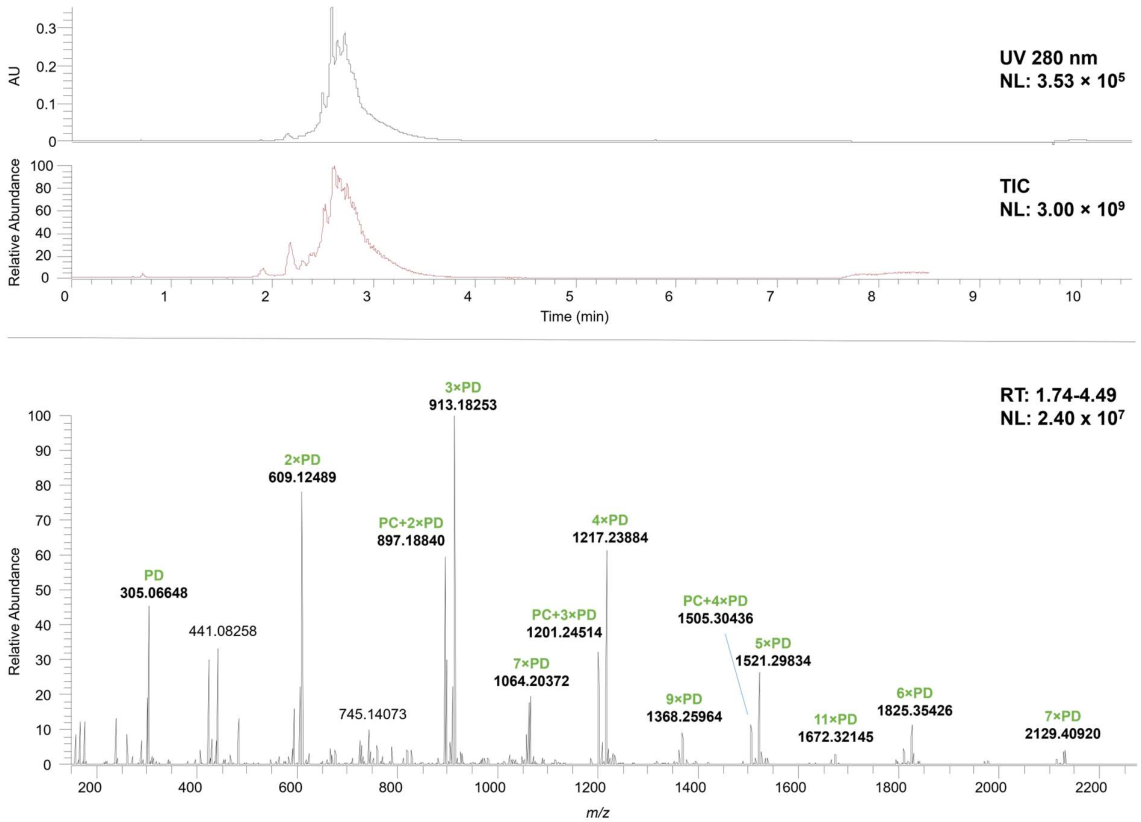

Figure S8. An UV chromatogram at 280 nm, total ion chromatogram (TIC) and a mass spectrum of the observed proanthocyanidin (PA) hump of the PA fraction B-PD 2 obtained from the ultra-high-resolution MS analysis. AU=absorbance unit, G=galloyl group, NL=normalised intensity, PC=procyanidin, PD=prodelphinidin, RT=retention time (min).

Table S8. Characterization of the main ions of the proanthocyanidin (PA) fraction B-PD 2 obtained from the ultrahigh-resolution MS analysis: the degree of polymerization (DP), molecular formulae, calculated exact mass, the monomeric composition of the PA oligomer and the molecular ion observed. PD=prodelphinidin.

DP	Molecular formula	Mcalculated	Monomeric composition	No. of A-type bonds	$[M-H]^-$	$[M-2H]^{2-}$
1	$C_{15}H_{14}O_7$	306.07396	PD	0	305.06648	-
2	$C_{30}H_{26}O_{14}$	610.13226	2×PD	0	609.12489	-
3	$C_{45}H_{38}O_{20}$	898.19565	PC+2×PD	0	897.18840	-
3	$C_{45}H_{38}O_{21}$	914.19057	3×PD	0	913.18235	-
4	$C_{60}H_{50}O_{27}$	1202.25396	PC+3×PD	0	1201.24514	-
4	$C_{60}H_{50}O_{28}$	1218.24887	4×PD	0	1217.23884	-
5	$C_{75}H_{62}O_{34}$	1506.31226	PC+4×PD	0	1505.30436	-
5	$C_{75}H_{62}O_{35}$	1522.30718	5×PD	0	1521.29834	-
6	$C_{90}H_{74}O_{41}$	1810.37057	PC+5×PD	0	1809.35866	-
6	$C_{90}H_{74}O_{42}$	1826.36548	6×PD	0	1825.35426	-
7	$C_{105}H_{86}O_{48}$	2114.42887	PC+6×PD	0	-	1056.20721
7	$C_{105}H_{86}O_{49}$	2130.42379	7×PD	0	2129.40920	-
7	$C_{105}H_{86}O_{49}$	2130.42379	7×PD	0	-	1064.20372
9	$C_{135}H_{110}O_{63}$	2738.54040	9×PD	0	-	1368.25964

11	$C_{165}H_{134}O_{77}$	3346.65701	11×PD	0	-	1672.32145

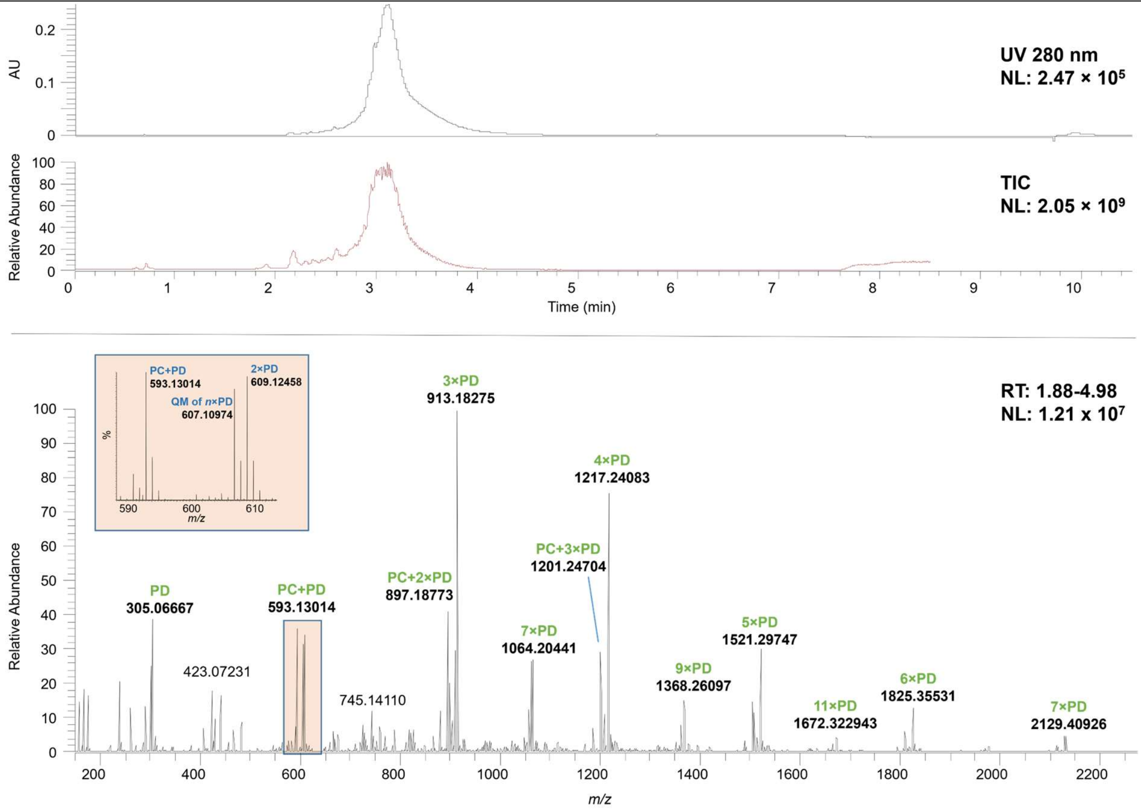

Figure S9. An UV chromatogram at 280 nm, total ion chromatogram (TIC) and a mass spectrum of the observed proanthocyanidin (PA) hump of the PA fraction B-PD 3 obtained from the ultra-high-resolution MS analysis. AU=absorbance unit, G=galloyl group, NL=normalised intensity, PC=procyanidin, PD=prodelphinidin, RT=retention time (min).

Table S9. Characterization of the main ions of the proanthocyanidin (PA) fraction B-PD 3 obtained from the ultrahigh-resolution MS analysis: the degree of polymerization (DP), molecular formulae, calculated exact mass, the monomeric composition of the PA oligomer and the molecular ion observed. PD=prodelphinidin and QM= quinone-methide cleavage.

DP	Molecular formula	Mcalculated	Monomeric composition	No. of A-type bonds	$[M-H]^-$	$[M-2H]^{2-}$
1	$C_{15}H_{14}O_7$	306.07396	PD	0	305.06667	-
2	$C_{30}H_{26}O_{13}$	594.13735	PC+PD	0	593.13014	-
2	$C_{30}H_{24}O_{14}$	608.11661	QM fragment of *n*×PD	0	607.10974	-
2	$C_{30}H_{26}O_{14}$	610.13226	2×PD	0	609.12458	-
3	$C_{45}H_{38}O_{20}$	898.19565	PC+2×PD	0	897.18773	-
3	$C_{45}H_{38}O_{21}$	914.19057	3×PD	0	913.18275	-
4	$C_{60}H_{50}O_{27}$	1202.25396	PC+3×PD	0	1201.24704	-
4	$C_{60}H_{50}O_{28}$	1218.24887	4×PD	0	1217.24083	-
5	$C_{75}H_{62}O_{34}$	1506.31226	PC+4×PD	0	1505.30424	-
5	$C_{75}H_{62}O_{35}$	1522.30718	5×PD	0	1521.29747	-
6	$C_{90}H_{74}O_{42}$	1826.36548	6×PD	0	1825.35531	-
7	$C_{105}H_{86}O_{49}$	2130.42379	7×PD	0	-	1064.20441
7	$C_{105}H_{86}O_{49}$	2130.42379	7×PD	0	2129.40926	-
9	$C_{135}H_{110}O_{63}$	2738.54040	9×PD	0	-	1368.26096

11	$C_{165}H_{134}O_{77}$	3346.65701	11×PD	0	-	1672.32294

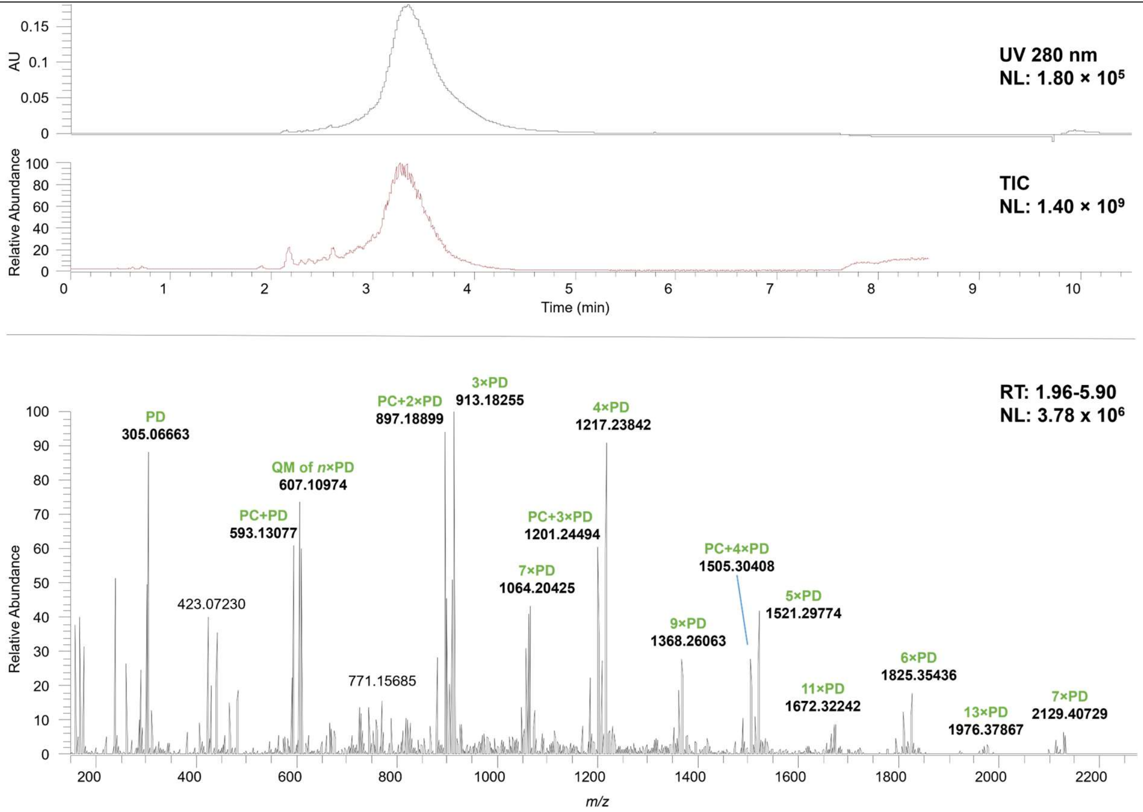

Figure S10. An UV chromatogram at 280 nm, total ion chromatogram (TIC) and a mass spectrum of the observed proanthocyanidin (PA) hump of the PA fraction B-PD 4 obtained from the ultra-high-resolution MS analysis. AU=absorbance unit, G=galloyl group, NL=normalised intensity, PC=procyanidin, PD=prodelphinidin, RT=retention time (min).

Table S10. Characterization of the main ions of the proanthocyanidin (PA) fraction B-PD 4 obtained from the ultrahigh-resolution MS analysis: the degree of polymerization (DP), molecular formulae, calculated exact mass, the monomeric composition of the PA oligomer and the molecular ion observed. PD=prodelphinidin and QM= quinone-methide cleavage.

DP	Molecular formula	Mcalculated	Monomeric composition	No. of A-type bonds	$[M-H]^-$	$[M-2H]^{2-}$
1	$C_{15}H_{14}O_7$	306.07396	PD	0	305.06663	-
2	$C_{30}H_{26}O_{13}$	594.13735	PC+PD	0	593.13077	-
2	$C_{30}H_{24}O_{14}$	608.11661	QM fragment	0	607.10974	-
2	$C_{30}H_{26}O_{14}$	610.13226	2×PD	0	609.12485	-
3	$C_{45}H_{38}O_{20}$	898.19565	PC+2×PD	0	897.18899	-
3	$C_{45}H_{38}O_{21}$	914.19057	3×PD	0	913.18255	-
4	$C_{60}H_{50}O_{27}$	1202.25396	PC+3×PD	0	1201.24494	-
4	$C_{60}H_{50}O_{28}$	1218.24887	4×PD	0	1217.23842	-
5	$C_{75}H_{62}O_{34}$	1506.31226	PC+4×PD	0	1505.30408	-
5	$C_{75}H_{62}O_{35}$	1522.30718	5×PD	0	1521.29774	-
6	$C_{90}H_{74}O_{41}$	1810.37057	PC+5×PD	0	1809.35845	-
6	$C_{90}H_{74}O_{42}$	1826.36548	6×PD	0	1825.35436	-
7	$C_{105}H_{86}O_{49}$	2130.42379	7×PD	0	-	1064.20425
7	$C_{105}H_{86}O_{49}$	2130.42379	7×PD	0	2129.40729	-

9	$C_{135}H_{110}O_{63}$	2738.54040	9×PD	0	-	1368.26063
11	$C_{165}H_{134}O_{77}$	3346.65701	11×PD	0	-	1672.32242
13	$C_{195}H_{158}O_{91}$	3954.77362	13×PD	0	-	1976.37867

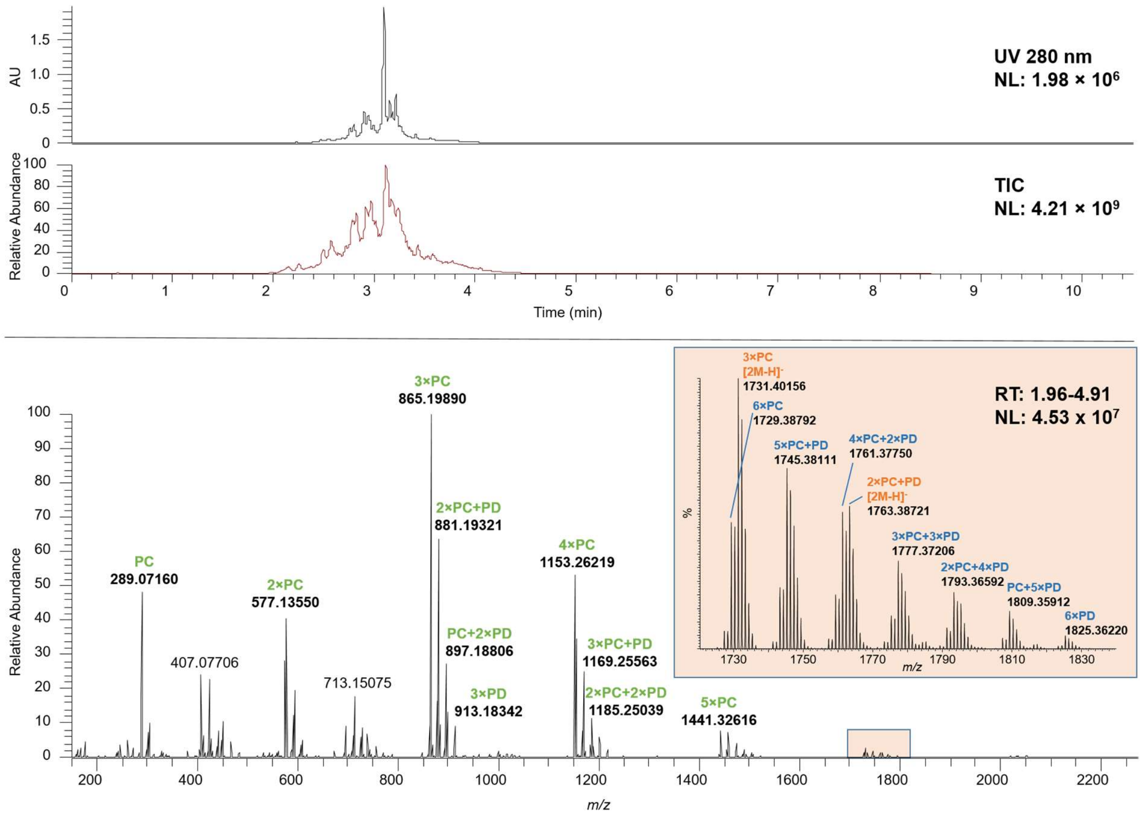

Figure S11. An UV chromatogram at 280 nm, total ion chromatogram (TIC) and a mass spectrum of the observed proanthocyanidin (PA) hump of the PA fraction B-PC 1 obtained from the ultra-high-resolution MS analysis. AU=absorbance unit, G=galloyl group, NL=normalised intensity, PC=procyanidin, PD=prodelphinidin, RT=retention time (min).

Table S11. Characterization of the main ions of the proanthocyanidin (PA) fraction B-PC 1 obtained from the ultrahigh-resolution MS analysis: the degree of polymerization (DP), molecular formulae, calculated exact mass, the monomeric composition of the PA oligomer and the molecular ion observed. PC=procyanidin and PD=prodelphinidin.

DP	Molecular formula	Mcalculated	Monomeric composition	No. of A-type bonds	$[M-H]^-$	$[2M-H]^-$
1	$C_{15}H_{14}O_6$	306.07396	PC	0	289.07160	-
2	$C_{30}H_{26}O_{12}$	578.14243	2×PC	0	577.13550	-
2	$C_{30}H_{26}O_{13}$	594.13735	PC+PD	0	593.13042	-
3	$C_{45}H_{38}O_{18}$	866.20582	3×PC	0	865.19890	-
3	$C_{45}H_{38}O_{18}$	866.20582	3×PC	0	-	1731.40156
3	$C_{45}H_{38}O_{19}$	882.20074	2×PC+PD	0	881.19321	-
3	$C_{45}H_{38}O_{20}$	898.19565	PC+2×PD	0	897.18806	-
4	$C_{60}H_{50}O_{24}$	1154.26921	4×PC	0	1153.26219	-
4	$C_{60}H_{50}O_{25}$	1170.26413	3×PC+PD	0	1169.25563	-
4	$C_{60}H_{50}O_{26}$	1186.25904	2×PC+2×PD	0	1185.25039	-

5	$C_{75}H_{62}O_{30}$	1442.33260	5×PC	0	1441.32616	-
5	$C_{75}H_{62}O_{31}$	1458.32752	PC+4×PD	0	1457.31864	-
5	$C_{75}H_{62}O_{32}$	1474.32243	2×PC+3×PD	0	1473.31270	-
6	$C_{90}H_{74}O_{36}$	1730.39599	6×PC	0	1729.38792	-

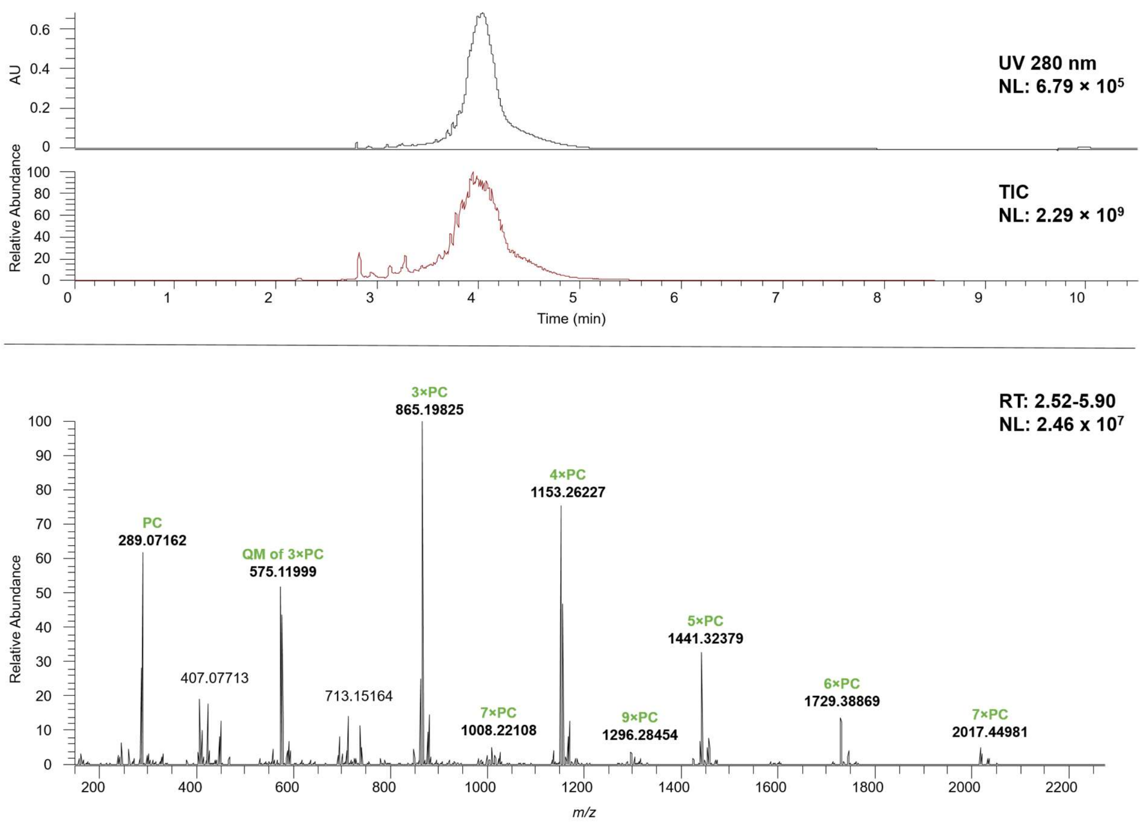

Figure S12. An UV chromatogram at 280 nm, total ion chromatogram (TIC) and a mass spectrum of the observed proanthocyanidin (PA) hump of the PA fraction B-PC 2 obtained from the ultra-high-resolution MS analysis. AU=absorbance unit, G=galloyl group, NL=normalised intensity, PC=procyanidin, PD=prodelphinidin, RT=retention time (min).

Table S12. Characterization of the main ions of the proanthocyanidin (PA) fraction B-PC 2 obtained from the ultrahigh-resolution MS analysis: the degree of polymerization (DP), molecular formulae, calculated exact mass, the monomeric composition of the PA oligomer and the molecular ion observed. PC=procyanidin, PD=prodelphinidin, QM= quinone-methide cleavage.

DP	Molecular formula	Mcalculated	Monomeric composition	No. of A-type bonds	$[M\text{-}H]^-$	$[M\text{-}2H]^{2-}$
1	$C_{15}H_{14}O_6$	290.07904	PC	0	289.07162	-
2	$C_{30}H_{24}O_{12}$	576.12678	QM fragment of 3×PC	0	575.11999	-
2	$C_{30}H_{26}O_{12}$	578.14243	2×PC	0	577.13529	-
3	$C_{45}H_{38}O_{18}$	866.20582	3×PC	0	865.19825	-
3	$C_{45}H_{38}O_{19}$	882.20074	2×PC+PD	0	881.19243	-
4	$C_{60}H_{50}O_{24}$	1154.26921	4×PC	0	1153.26227	-
4	$C_{60}H_{50}O_{25}$	1170.26413	3×PC+PD	0	1169.25529	-
5	$C_{75}H_{62}O_{30}$	1442.33260	5×PC	0	1441.32379	-
5	$C_{75}H_{62}O_{31}$	1458.32752	4×PC+PD	0	1457.31656	-

6	$C_{90}H_{74}O_{36}$	1730.39599	6×PC	0	1729.38869	-
7	$C_{105}H_{86}O_{42}$	2018.45938	7×PC	0	2017.44981	-
7	$C_{105}H_{86}O_{42}$	2018.45938	7×PC	0	-	1008.22108
9	$C_{135}H_{110}O_{54}$	2594.58616	9×PC	0	-	1296.28454

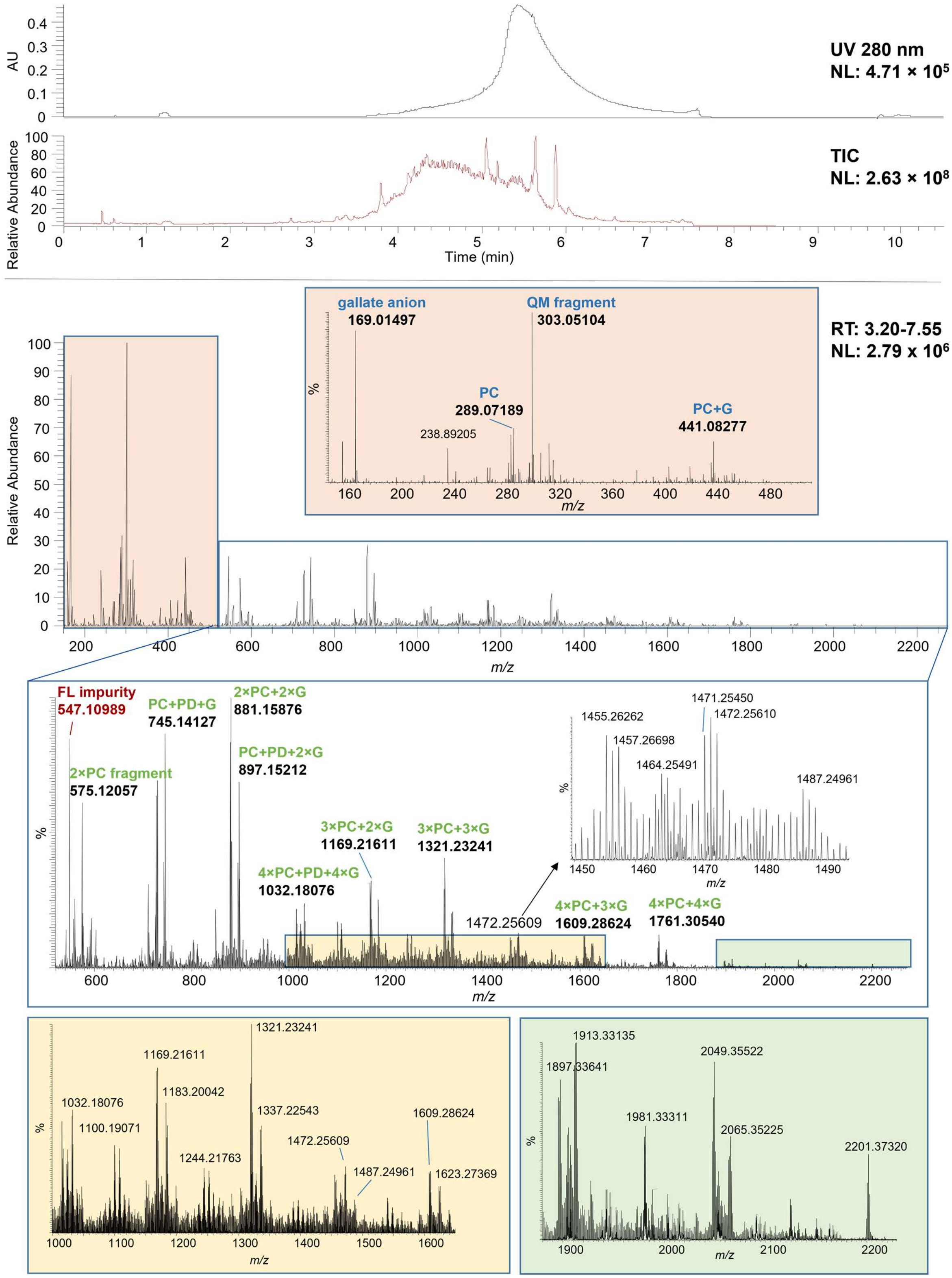

Figure S13. An UV chromatogram at 280 nm, total ion chromatogram (TIC) and a mass spectrum of the observed proanthocyanidin (PA) hump of the PA fraction B-PC-G 2 obtained from the ultra-high-resolution MS analysis. AU=absorbance unit, FL=flavonoid, G=galloyl group, NL=normalised intensity, PC=procyanidin, PD=prodelphinidin, RT=retention time (min).

Table S13. Characterization of the main ions of the proanthocyanidin (PA) fraction B-PC-G 2 obtained from the ultrahigh-resolution MS analysis: the degree of polymerization (DP), molecular formulae, calculated exact mass, the monomeric composition of the PA oligomer and the molecular ion observed. FL=flavonoid, G=galloyl group, PC=procyanidin, PD=prodelphinidin, QM= quinone-methide cleavage, RDA= retro-Diels-Alder.

DP	Molecular formula	Mcalculated	Monomeric composition	$[M-H]^-$	$[M-2H]^{2-}$
-	$C_7H_6O_5$	170.02153	gallate anion	169.01497	-
1	$C_{15}H_{14}O_6$	290.07904	PC	289.07189	-
1	$C_{15}H_{12}O_7$	304.05831	QM fragment	303.05104	-
1	$C_{22}H_{18}O_{10}$	442.09000	PC+G	441.08277	-
-	$C_{25}H_{24}O_{14}$	548.11661	FL impurity	547.10989	-
2	$C_{30}H_{24}O_{12}$	576.12678	2×PC (fragment of *m*/*z* 727)	575.12057	-
2	$C_{37}H_{28}O_{16}$	728.13774	2×PC+G (fragment of *m*/*z* 879)	727.13144	-
2	$C_{37}H_{30}O_{16}$	730.15339	2×PC+G	729.14687	-
2	$C_{37}H_{30}O_{17}$	746.14831	PC+PD+G	745.14127	-
2	$C_{45}H_{36}O_{19}$	880.18509	2×PC+2×G (fragment of higher oligomer)	879.17543	-
2	$C_{44}H_{34}O_{20}$	882.16435	2×PC+2×G	881.15876	-
2	$C_{44}H_{34}O_{21}$	898.15927	PC+PD+2×G	897.15212	-
3	$C_{59}H_{46}O_{26}$	1170.22774	3×PC+2×G	1169.21611	-
3	$C_{59}H_{44}O_{27}$	1184.20701	2×PC+PD+2×G (fragment of higher oligomer)	1183.20042	-
3	$C_{66}H_{50}O_{30}$	1322.23870	3×PC+3×G	1321.23241	-
3	$C_{66}H_{50}O_{31}$	1338.23362	2×PC+PD+3×G	1337.22543	-
4	$C_{81}H_{62}O_{36}$	1610.30209	4×PC+3×G	1609.28624	-
4	$C_{81}H_{60}O_{37}$	1624.28136	3×PC+PD+3×G	1623.27369	-
4	$C_{88}H_{66}O_{40}$	1762.31305	4×PC+4×G	1761.30540	-
4	$C_{88}H_{66}O_{41}$	1778.30797	3×PC+PD+4×G	1777.29936	-
4	$C_{74}H_{56}O_{32}$	1456.27548	4×PC+2×G (fragment of higher oligomer)	1455.26262	-
4	$C_{74}H_{56}O_{33}$	1472.27040	3×PC+PD+2×G (fragment of higher oligomer)	1471.25450	-
4	$C_{74}H_{56}O_{34}$	1488.26531	2×PC+2×PD+2×G (fragment of higher oligomer)	1487.24961	-
5	$C_{95}H_{70}O_{43}$	1898.32910	RDA fragment of 5×PC+4×G	1897.33641	-
5	$C_{95}H_{70}O_{44}$	1914.32401	RDA fragment of 4×PC+PD+4×G	1913.33135	-
5	$C_{103}H_{78}O_{46}$	2050.37644	5×PC+4×G	2049.35522	-
5	$C_{110}H_{82}O_{50}$	2202.38740	5×PC+5×G	2201.37320	-
5	$C_{103}H_{78}O_{47}$	2066.37136	4×PC+PD+4×G	-	1032.18076
5	$C_{110}H_{82}O_{50}$	2202.38740	5×PC+5×G	-	1100.19071
5	$C_{103}H_{78}O_{47}$	2066.37136	4×PC+PD+4×G	2065.35225	-
6	$C_{125}H_{94}O_{56}$	2490.45079	6×PC+5×5	-	1244.21763

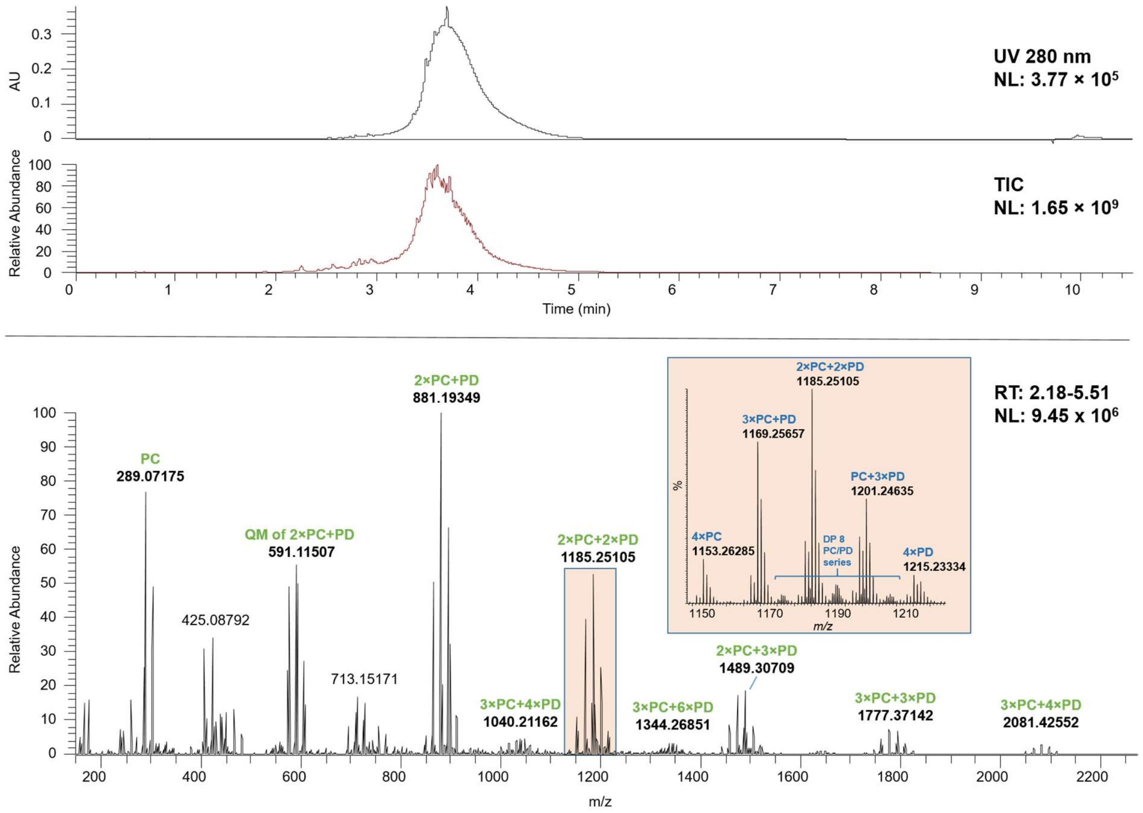

Figure S14. An UV chromatogram at 280 nm, total ion chromatogram (TIC) and a mass spectrum of the observed proanthocyanidin (PA) hump of the PA fraction B-PC/PD obtained from the ultra-high-resolution MS analysis. AU=absorbance unit, G=galloyl group, NL=normalised intensity, PC=procyanidin, PD=prodelphinidin, RT=retention time (min).

Table S14. Characterization of the main ions of the proanthocyanidin (PA) fraction B-PC/PD obtained from the ultrahigh-resolution MS analysis: the degree of polymerization (DP), molecular formulae, calculated exact mass, the monomeric composition of the PA oligomer and the molecular ion observed. PC=procyanidin, PD=prodelphinidin, QM= quinone-methide cleavage.

DP	Molecular formula	Mcalculated	Monomeric composition	No. of A-type bonds	$[M\text{-}H]^-$	$[M\text{-}2H]^{2-}$
1	$C_{15}H_{14}O_6$	290.07904	PC	0	289.07175	-
1	$C_{15}H_{14}O_7$	306.07396	PD	0	305.06663	-
2	$C_{30}H_{26}O_{12}$	578.14243	2×PC	0	577.13593	-
2	$C_{30}H_{24}O_{13}$	592.12170	QM fragment of 2×PC+PD	0	591.11507	-
2	$C_{30}H_{26}O_{13}$	594.13735	PC+PD	0	593.13036	-
2	$C_{30}H_{24}O_{14}$	608.11661	QM fragment	0	607.10927	-
3	$C_{45}H_{38}O_{18}$	866.20582	3×PC	0	865.19912	-
3	$C_{45}H_{38}O_{19}$	882.20074	2×PC+PD	0	881.19349	-
3	$C_{45}H_{36}O_{20}$	896.18000	QM fragment	0	895.17210	-
3	$C_{45}H_{38}O_{20}$	898.19565	PC+2×PD	0	897.18790	-
4	$C_{60}H_{50}O_{25}$	1170.26413	3×PC+PD	0	1169.25657	-
4	$C_{60}H_{50}O_{26}$	1186.25904	2×PC+2×PD	0	1185.25105	-
4	$C_{60}H_{50}O_{27}$	1202.25396	PC+3×PD	0	1201.24635	-
5	$C_{75}H_{62}O_{31}$	1458.32752	4×PC+PD	0	1457.31759	-
5	$C_{75}H_{62}O_{32}$	1474.32243	3×PC+2×PD	0	1473.31401	-

5	$C_{75}H_{62}O_{33}$	1490.31735	2×PC+3×PD	0	1489.30709	-
6	$C_{90}H_{74}O_{39}$	1778.38074	3×PC+3×PD	0	1777.37142	-
6	$C_{90}H_{74}O_{40}$	1794.37565	2×PC+4×PD	0	1793.3654	-
7	$C_{105}H_{86}O_{46}$	2082.43904	3×PC+4×PD	0	2081.42552	-
7	$C_{105}H_{86}O_{46}$	2082.43904	3×PC+4×PD	0	-	1040.21162
9	$C_{135}H_{110}O_{60}$	2690.55565	3×PC+6×PD	0	-	1344.26851

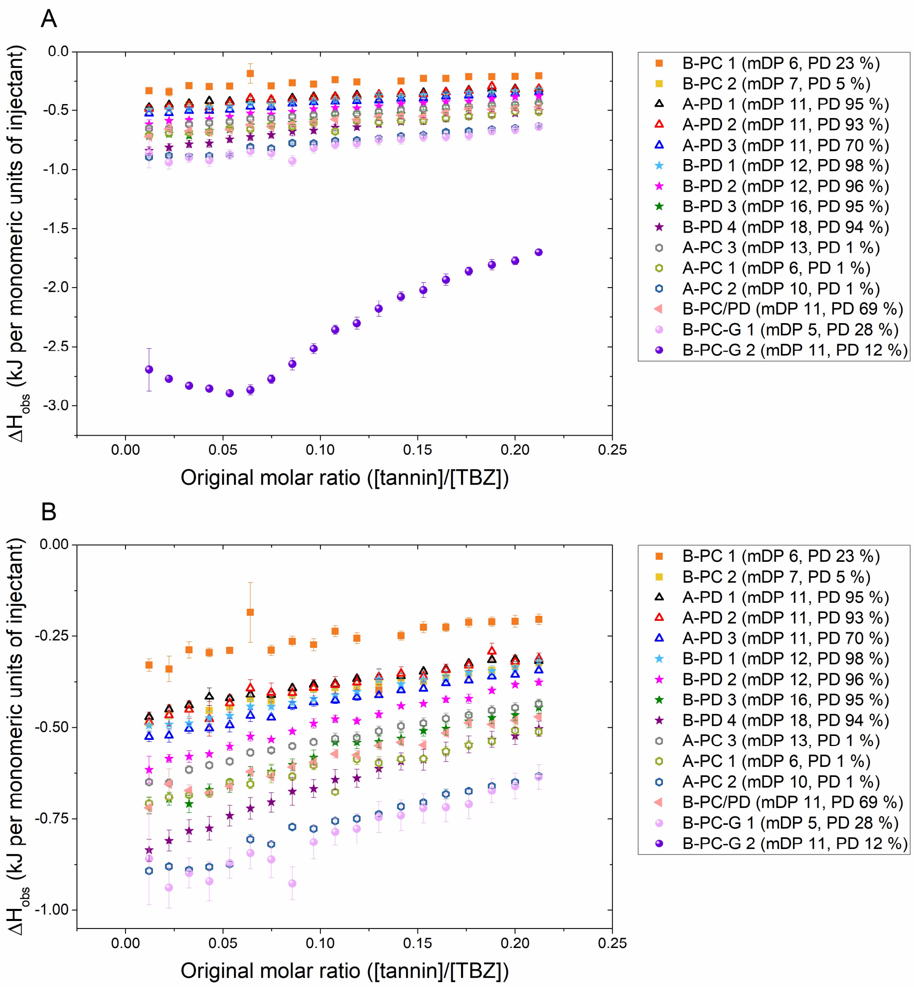

Figure S15. Comparison of observed enthalpies (ΔH_{obs}) from the proanthocyanidin fractions with the effect of the degree of polymerization taken into account (A) and a close-up on the smaller enthalpy changes (B). Fractions from the same plant source are marked in the same symbols and fractions containing A-type linkages are marked with symbols with an open center. For an explanation of the fraction codes, please refer to the original article.
